# COVID-19-Related Age Profiles for SARS-CoV-2 Variants in England and Wales and States of the USA (2020 to 2022): Impact on All-Cause Mortality

**DOI:** 10.3390/idr15050058

**Published:** 2023-10-08

**Authors:** Rodney P. Jones, Andrey Ponomarenko

**Affiliations:** 1 Healthcare Analysis & Forecasting, Wantage OX12 0NE, UK; 2 Department of Biophysics, Informatics and Medical Instrumentation, Odessa National Medical University, Valikhovsky Lane 2, 65082 Odessa, Ukraine

**Keywords:** COVID-19, all-cause mortality, year-of-age, sex, complex system, SARS-CoV-2 variants, age-specificity, birth cohorts, miRNAs

## Abstract

Since 2020, COVID-19 has caused serious mortality around the world. Given the ambiguity in establishing COVID-19 as the direct cause of death, we first investigate the effects of age and sex on all-cause mortality during 2020 and 2021 in England and Wales. Since infectious agents have their own unique age profile for death, we use a 9-year time series and several different methods to adjust single-year-of-age deaths in England and Wales during 2019 (the pre-COVID-19 base year) to a pathogen-neutral single-year-of-age baseline. This adjusted base year is then used to confirm the widely reported higher deaths in males for most ages above 43 in both 2020 and 2021. During 2020 (+COVID-19 but no vaccination), both male and female population-adjusted deaths significantly increased above age 35. A significant reduction in all-cause mortality among both males and females aged 75+ could be demonstrated in 2021 during the widespread COVID-19 vaccination period; however, deaths below age 75 progressively increased. This finding arises from a mix of vaccination coverage and year-of-age profiles of deaths for the different SARS-CoV-2 variants. In addition, specific effects of age around puberty were demonstrated, where females had higher deaths than males. There is evidence that year-of-birth cohorts may also be involved, indicating that immune priming to specific pathogen outbreaks in the past may have led to lower deaths for some birth cohorts. To specifically identify the age profile for the COVID-19 variants from 2020 to 2023, we employ the proportion of total deaths at each age that are potentially due to or ‘with’ COVID-19. The original Wuhan strain and the Alpha variant show somewhat limited divergence in the age profile, with the Alpha variant shifting to a moderately higher proportion of deaths below age 84. The Delta variant specifically targeted individuals below age 65. The Omicron variants showed a significantly lower proportion of overall mortality, with a markedly higher relative proportion of deaths above age 65, steeply increasing with age to a maximum around 100 years of age. A similar age profile for the variants can be seen in the age-banded deaths in US states, although they are slightly obscured by using age bands rather than single years of age. However, the US data shows that higher male deaths are greatly dependent on age and the COVID variant. Deaths assessed to be ‘due to’ COVID-19 (as opposed to ‘involving’ COVID-19) in England and Wales were especially overestimated in 2021 relative to the change in all-cause mortality. This arose as a by-product of an increase in COVID-19 testing capacity in late 2020. Potential structure–function mechanisms for the age-specificity of SARS-CoV-2 variants are discussed, along with potential roles for small noncoding RNAs (miRNAs). Using data from England, it is possible to show that the unvaccinated do indeed have a unique age profile for death from each variant and that vaccination alters the shape of the age profile in a manner dependent on age, sex, and the variant. The question is posed as to whether vaccines based on different variants carry a specific age profile.

## 1. Introduction

The SARS-CoV-2 pandemic has caused considerable human morbidity and mortality, necessitating studies to develop clinical protocols, repurpose existing drugs, and develop vaccines to counter these adverse effects [1]. The pandemic has seen a progression of new variants emerge in different parts of the world that appear to have different age profiles for death [1,2]—although the precise nature of the age profile for death is obscured by the fact that different studies use a wide range of age bands ending at age 75+ through to 90+ [3,4,5,6,7]. Influenza and SARS-CoV-2 are both examples of a class of RNA viruses showing high mutation rates [8,9,10,11,12], hence the regular emergence of new clinically significant variants.

In the UK, the COVID-19 pandemic commenced somewhere in early 2020, with the first laboratory-confirmed death occurring on 2 March 2020 [13]. However, COVID-19 testing capacity in the UK was very low at that time, and earlier deaths due to COVID-19 were possible [14]. 

Research in the USA suggests that COVID-19 deaths may have started in early January 2020 [15]. A general paucity of testing capacity in the UK during the first year of the pandemic [14] implies that all-cause mortality becomes a good proxy for the incremental changes in real COVID-19 mortality [16]. 

In both the UK and USA, the original strain was predominant in 2020. The Alpha strain appeared around October/November 2020 and was predominant from January to June 2021; the Delta strain commenced around May 2021 and was predominant from July to December 2021. While Omicron first emerged in November 2021, it began to spread in December 2021 and was dominant in 2022 from around March onward [17,18]. The Beta strain did not appear to gain a major foothold in these two countries [16,17]. Hence, we have the pre-COVID-19 era, which ended in December 2019, through to the ongoing surges as new variants came to the fore from the end of 2020 onward [17,18].

It is important to recall that deaths are a lagging indicator since infection, illness, and hospitalization precede recovery or death. The gap between infection and death is highly likely to have a probability distribution that depends on age and risk factors [19]. The Alpha variant caused slightly higher mortality than the original strain and primarily affected mortality in the winter of 2020/21. The Delta variant mainly affected the winter of 2021/22 with higher transmission and a slightly lower or equal mortality risk [20,21,22]. 

In the UK, COVID-19 vaccines were approved and deployed in the following order: Pfizer/BioNTech (2 December 2020—deployed 8 December 2020), AstraZeneca (30 December 2020—deployed 4 January 2021), and Moderna (8 January 2021—deployed 7 April 2021) [23,24,25,26]. Data regarding the proportions of persons vaccinated by age and time with the different manufacturers (Pfizer/AstraZeneca/Moderna) are not publicly available.

COVID-19 vaccination began on 8 December 2020 for care home residents, persons aged 80+, and some health care workers; by 18 January 2021 this included persons aged 70+ and persons with very high clinical risk; by 15 February, persons aged 65+ and persons with high risk; by 22 May, persons aged 32+; and persons aged 18+ by 18 June 2021 [23,24,25,26]. Following reports of a rare type of blood clot in late March 2021 for the AstraZeneca vaccine, persons under 30 years were all given the mRNA vaccine from 7 April 2021 onward, and those aged under 40 from 7 May 2021 onward [26]. Vaccination of persons aged 16–17 years was from July 2021 onward, 12–15 years from September 2021 onward, and 5–11 years from February 2022 onward. The majority of those aged 12+ were vaccinated in late 2021 with the mRNA vaccine, as per the age-under-40 rule from above. From around spring 2022 onward, all persons were vaccinated (including boosters) mainly with the mRNA vaccine [23,24,25,26], while in spring 2022, a non-mRNA (Novavax recombinant protein) was introduced to replace the AstraZeneca vaccine, which was withdrawn around mid-2021 [23,24,25,26].

As mentioned above, studies on the effects of age generally use wide age bands, which can conceal the underlying year-of-age behavior. A World Bank analysis of the COVID-19-related mortality rate during the first wave of COVID-19 in 2020 used 10-year age bands commencing at 30–39 through to age 80+ to demonstrate typical logarithmic decay in the mortality rate as age reduces. The World Bank study notes that the mortality rate in low-income countries is flatter than in high-income countries, with up to a 10-times higher mortality rate at age 30–39 [3]. 

Another study using 2020 data shows a similar log-decay and estimates that the COVID-19 mortality rate, relative to the average for influenza and pneumonia (1999–2018), is five-times higher at age 25–34, eight-times higher at 55–64, but only three-times higher at 85+ [4]. Adjei et al. [5] compare in-hospital mortality rates of the Delta and Omicron variants using 15-year age bands, 0–17, through to 80+. Mortality rates for the age band 0–17 are Delta/Omicron 0.7/0.9 through to 21.6/6.5 at age 80+. Stepanova et al. [6] used 10-year age bands commencing at age < 55 years through to age 75+ when studying the effects of vaccination upon hospitalization and death from the Alpha, Delta, and Omicron variants [6]. Leiner et al. [7] use age bands of < 59 years, 60–69, 70–79, and 80+ to adjust hospital outcomes from the Delta and Omicron variants [7]. Without detailing all studies, there is gross variation between age bands and hidden assumptions regarding behavior within age bands. Arbitrary age bands can cut across, and so conceal, underlying trends. 

An excellent example of the potential for age bands to conceal relevant behavior was the large spike in deaths during the 1918–1919 Spanish flu pandemic at the precise age of 28, due to exposure of this birth cohort to the previous 1889–1890 Russian flu pandemic. Seemingly, this birth cohort was ‘vaccinated’ due to natural exposure to the Russian flu, which had adverse outcomes when later exposed to the antigenically dissimilar Spanish flu [27]. 

As far as we are aware, there are no detailed single-year-of-age studies for SARS-CoV-2 variants, and this study therefore seeks to establish single-year-of-age profiles for the deaths arising from different variants that predominated in England and Wales between 2020 and 2022. These will be compared to age-banded results for the USA and US states. A method based on the proportion of total all-cause deaths either ‘with’ or ‘due to’ COVID-19 is used to separate the specific effects against age per se.

We first use year-of-age calendar year data for 2019 to 2022 for England and Wales covering all-cause mortality, which has the advantage of containing more than 530,000 deaths per annum. We then turn to monthly analysis to refine the age profiles for deaths arising from different COVID-19 variants. 

## 2. Materials and Methods

### 2.1. Data Sources

Year-of-age all-cause [28,29] and ‘caused by’ COVID-19 deaths in England and Wales are from the Office for National Statistics (ONS) [30,31]. Monthly deaths in England and Wales are from the ONS [32]. Year-of-age population estimates for 2011–2020 are from the ONS [33]. Weekly deaths in the UK are reported by the ONS [34]. 

The year-of-age male and female populations for 2021 were estimated using the method described in Section 2.3. Deaths by vaccination status are also reported by the ONS [35]. Annual births in England and Wales are also from the ONS [36]. Monthly data covering ‘with’ COVID-19 deaths and all-cause mortality from the USA is from the US Centers for Disease Control and Prevention (CDC) [37]. The CDC masks all state-level deaths between 1 and 9, hence all blanks were replaced with 1, and the likely value was interpolated by the ratio of total COVID-19 deaths in the peak variant month to the month with missing values. Any value of this ratio > 9 is limited to a value of 9. National deaths in the USA are not masked in this way. An analysis was mainly conducted for the larger states, where very little data needed to be masked.

Finally, population forecasts (including deaths) in England are from the ONS [38].

### 2.2. Date at Which a Death Is Reported Versus Date of Occurrence

As in all countries, deaths in England and Wales are recorded as the date on which the death is registered rather than the date on which the death occurred. Depending on the disposition of public holidays at the end/beginning of calendar years, this can lead to a 0.3% to 0.5% difference in the number of registered deaths. The difference between years in which deaths occurred carried forward at the beginning and end of the year is usually reasonably well matched [39]. As long as the method remains unchanged, such differences do not have a material impact on this study. The effect on monthly data is likewise minimized due to the data being summed over the multiple months during which each SARS-CoV-2 variant predominated.

### 2.3. Estimation of the Year-of-Age Population in 2021

The estimates of population between the decennial censuses represent a potential for bias. The census-based 2021 population was 1.1% lower than the 2018-based forecast. However, this occurs mostly among working-age younger adults due to inward/outward immigration between England and Wales and the European Union’s Eastern European countries [38]. Once again, this does not have a material impact on this study due to the very low COVID-19 deaths in these age groups.

Due to the 2021 Census, the release of the 2021 population estimates for England and Wales has been delayed to 2023 and was not available for this study. However, using 2020 forecast population data and 2021 deaths can give an estimate with good accuracy. The method is straightforward. Living persons of any age move forward by one year of age each year. Take this number and then subtract the number of deaths. This was performed between 2011 and 2020. 

This can be called a population aging model without net migration. This will be an underestimate due to net inward immigration. Inward immigration can be inferred from the difference between the result of this method and the ONS estimates. Net inward immigration is highest in the younger age groups; hence, in 2020, the population of 20-year-old males/females was estimated to be 2.7% and 2.0% higher, respectively, than the simple population aging model, while that for 1-year-old males/females was 1.1% higher for both genders. In 2020, the net inward migration component was less than 0.3% per year for all ages above 32. The 2021 population was calculated using this net migration-adjusted method. This is very similar to the ONS model.

### 2.4. Estimation of the Year-of-Age Baseline for Deaths in England and Wales in 2019

Actuarial forecasts for future deaths are notoriously unreliable (see Supplementary Material S1 [16,38,40,41]), mainly due to high uncertainty in the future mortality rate. These annual numbers show high volatility (as shown in Supplementary Material S1), largely due to winter infectious outbreaks [40,41], which in the Northern Hemisphere span two calendar years. 

Most government statistical agencies use the average of the previous five years to estimate the baseline [31], which from Supplementary Material S1 can be seen to be subject to bias depending on whether deaths are falling (through to 2011 in Supplementary Material S1) or rising (after 2011 in Supplementary Material S1). The falling/rising patterns in death depend on past demographic shifts (mainly births and immigration) and are particular to each country.

The year 2019 represents the pre-COVID-19 baseline for mortality. The raw deaths in 2019 for England and Wales are available [28,29]. However, they are subject to both Poisson-based and systematic variation due largely to infectious outbreaks [40,41] and the occasional prolonged heatwave or prolonged excessive cold—note that the emphasis is on prolonged, or above and beyond a normal summer/winter [42,43]. Each source of volatility will have its own age profile. It is therefore necessary to adjust the actual 2019 deaths to give an estimate of the 2019 baseline, which would be the case had infectious and other age-specific events been at ‘the average’ in that year. This estimation was performed using five different methods. In all cases, actual deaths for the 8 years (2011–2018) were converted to an adjusted 2019 equivalent. The adjustment methods are detailed in the Supplementary Materials S2 and S3. All adjusted deaths, including those in 2019, were then extrapolated to 2019 using a second-order polynomial curve fit (Microsoft Excel—Add trendline function). The adjustment factor varied with the method of adjustment, namely, population, prior births (two alternative methods), raw deaths—no adjustment, or the average of 2017 to 2019. Table S4 provides the resulting estimates for the 2019 baseline deaths, along with an analysis of the standard deviation associated with each age of death. All methods gave tightly clustered estimates for 2019 baseline deaths up to age 70 in both males and females. The highest standard deviation for the estimates occurred at age 88 (± 3.3%) in males or age 93 (± 4.5%) in females.

Firstly, raw deaths between 2011 and 2018 [28] were converted to a 2019 equivalent by multiplying by the 2019 mid-year year-of-age estimated population divided by that in each of the years from 2011 to 2018. Given that human mortality is changing over time [44], both linear and second-order polynomial regression were applied to these adjusted deaths to give the most likely average for 2019, which will be relatively free of both Poisson and systematic variation. Visual inspection showed that a second-order polynomial usually gave the best estimate for the ‘average’ or ‘baseline’ deaths in 2019—although in most instances the actual differences are minor. This is consistent with the observation that there was a breakpoint in international mortality improvement from 2011 onward [44]. Exceptions include infants in the first year of life, where population-adjusted male and female mortality was constant between 2013 and 2019. 

Due to high uncertainty in the population of persons aged 90+, the ONS reports the population of 90+ persons as a single group [28,29]. In 2019, this includes persons born in 1929 (age 90) and before and will include the increase in population numbers due to the World War I baby boom occurring in England and Wales [35]. 

We note that the WWI baby boom affected some countries far less than the UK. While most statistical agencies avoid analysis of the 100+ age group, we nevertheless include such analysis given the potential for birth cohort effects, which are discussed later. WWI was associated with the Spanish flu pandemic [27,45], and the surviving children and newborns represent a potential group of interest.

Year-of-age deaths for ages above 90 were estimated based on either a second-order polynomial regression of actual deaths between 2011 and 2019 or an adjustment based on the number of births [35]. These ages are complicated by the presence of the World War I baby boom (see Supplementary Materials S2 and S3 [28,29,40,41,45]). For adjustment based on births, the number of births is substituted for the population (as above), with all years adjusted to the 2019 equivalent. Baseline deaths in 2019 were also estimated using a second-order polynomial based on raw deaths between 2011 and 2019 (ages 80 to 105+) and using the average of deaths in 2018 and 2019. The rationale for this last adjustment is given in Supplementary Materials S2 and S3. A large infectious event in early 2018 with an associated age profile necessitated an additional method based on the average deaths from 2017 to 2019. The final estimate for the 2019 baseline was derived from the average of the 3 or 4 different methods, depending on age, and is shown in Supplementary Material S4.

The Poisson-based standard deviation (STDEV) associated with deaths, as a percentage value, was calculated as the square root of the number of deaths divided by the number of deaths. The variation associated with the difference between male and female deaths was calculated as the STDEV for males plus the STDEV for females—as a percentage value.

**Figure A1:**
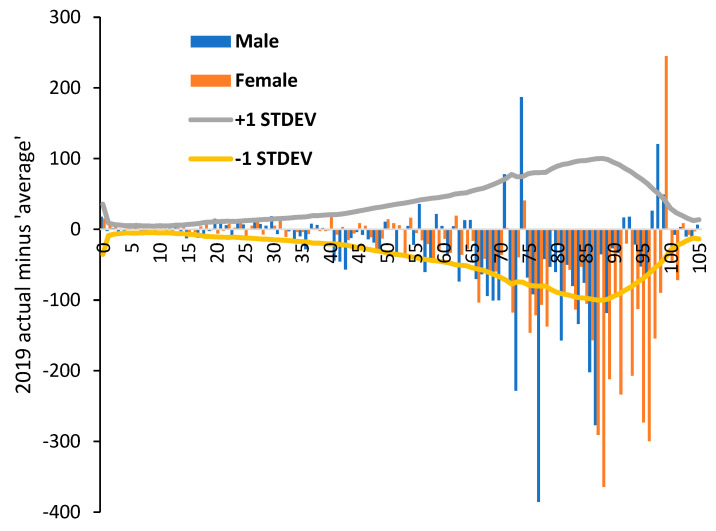
Difference between the actual 2019 deaths and the underlying trend of deaths (the ‘average’) in 2019, by single-year-of-age in England and Wales. STDEV = standard deviation (Poisson).

The difference between actual deaths in 2019 and the estimated baseline, or ‘average’, for 2019 is shown in Figure A1 in Appendix A, where in the younger ages, the difference between actual and baseline is usually within ± 1 standard deviation; however, above age 70, it is consistently below −1 standard deviation. Hence, it is in the older age groups that 2019 deaths are low. See Supplementary Material S2 for an explanation. Several other ages show systematic deviation, namely, males aged 41–43, 57, 63, 66–70, and 73 (all low), ages 74 and 98 (both high), and females aged 99 (high).

### 2.5. Calculation of the Proportion of Deaths ‘With’ or ‘Due to’ COVID-19 Variants

Inspection of the monthly raw data for England and Wales and US states shows approximately similar timing in the occurrence of deaths from the different variants. Hence, the following time periods are used to aggregate both COVID-19 deaths (numerator) and all-cause mortality (denominator): original Wuhan strain (March 2020 to September 2020), Alpha variant (October 2020 to June 2021), Delta variant (July 2021 to February 2022), and Omicron variant (March 2022 onward).

## 3. Results

It is very common for government statistical agencies to present detailed analyses of deaths from one calendar year to the next. We first present an analysis using annual year-of-age data in England and Wales for 2019 (corrected as above) versus 2020 and 2021. We then move to a detailed monthly analysis of the highly specific age profiles for the various SARS-CoV-2 variants and how these may impact the results seen in various calendar years. This study has a focus on all-cause mortality simply because assessing a genuine COVID-19-induced death appears to be subject to international ambiguity [16]—see Supplementary Materials S5 [46,47,48,49,50,51].

### 3.1. Validation of Deaths ‘Due to’ COVID-19 and Methods for Adjusting Deaths to a Common Year

This study used two primary data sources:

1. Monthly deaths for all-cause and ‘due to’ COVID-19 mortality by year-of-age [30]—not split by male and female, but does cover 2022.
2. Annual all-cause deaths by year-of-age [28,29]—has the benefit of large numbers and is split by male and female, but only covers COVID-19 years 2020 and 2021.

These two sources were used to cross-check each other, and the full details are given in Supplementary Materials S2–S5 [38,39,40,41,42,43,44,45,46,47,48,49,50,51].

The main conclusions are as follows:

1. Adjusting deaths to the 2019 equivalent using mid-year population estimates contains hidden assumptions regarding the monthly profile of the influx of births during the World War I and II baby booms, and an alternative adjustment using annual births can be utilized.
2. The matching between population/births and deaths also contains assumptions around the pattern of seasonal deaths. This is not an issue in the southern hemisphere, where winter occurs in the middle of a calendar year, but it creates inconsistencies in the northern hemisphere since winter spans two calendar years.
3. The age profiles for excess mortality (with various adjustments) versus COVID-19 ‘due to’ deaths are roughly similar; however, deaths ‘due to’ COVID-19 appear to have been over-estimated, especially in 2021 among elderly females (with vaccination)—reflecting the well-known difficulty of attributing cause of death in elderly persons with multi-morbidities along with other factors [16]. See Supplementary Material S5.
4. No discernible impact of COVID-19 upon all-cause deaths can be observed for ages 100+ or ages 1–30 either with or without adjustment—partly due to small number variation as in Supplementary Material S2.

Regardless of the sources of variation in monthly deaths, it is concluded that the method based on the proportion (%) of monthly all-cause deaths that are assessed as either ‘with’ or ‘due to’ COVID-19 is reliable for the estimation of year-of-age profiles for COVID-19 deaths. This is because both total all-cause deaths and assessed COVID-19 deaths in a month or collection of months are always matched. Since it is a proportion, this method is independent of differences in population numbers between ages.

### 3.2. Population-Adjusted Deaths in 2020 and 2021 versus 2019

**Figure 1:**
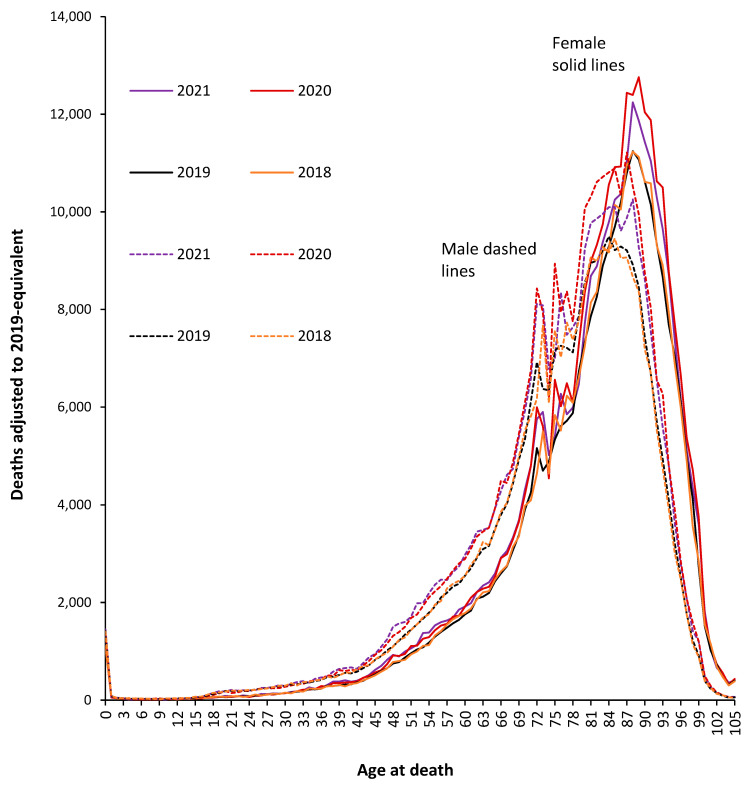
Single-year-of-age-adjusted all-cause deaths in England and Wales for males and females between 2018 and 2021. Data for 2019 are the ‘average’ expected (the baseline) for that year based on the trend between 2011 and 2019. Age concludes at 105+.

The first waves of the COVID-19 pandemic during 2020 were especially hard and made worse by the absence of any vaccine, repurposed drugs, or clinical protocols [1,2]. Figure 1 therefore illustrates this effect for males and females during both 2020 and 2021 versus the baseline deaths in 2019—as in Supplementary Material S2—with 2018 included for additional comparison. 

From Figure 1, the pre-COVID-19 age profile is more peaked for females, and the absolute number of deaths reaches a higher maximum in females, peaking at 89 in females compared to 84 in males. Certain ages are exaggerated during COVID-19, while the two pre-COVID-19 years (2018 and the 2019 ‘adjusted’) are very close together. It should be noted that the use of 5-year or 10-year age bands would completely obscure the unique behavior at specific ages. Two sub-peaks around ages 72 and 75 are the outcome of various baby booms around World War II [36]. The age 72 sub-peak is after the war finishes, while the age 75 sub-peak arose from final visits home for soldiers departing for the D-Day landings. However, by far the greatest absolute number of deaths occur above age 78, which is the outcome of a mid-WWII mini-baby boom (see Figure S4 in the Supplementary Materials), which in theory should represent the priority target population for vaccination—see further discussion later. Such nuances will be specific to England and Wales and will vary depending on the population age distribution.

As can be seen, all-cause deaths in 2020 (without vaccination) were especially higher for males aged over 50 years, while in females this is not the case until above age 78. In 2021 (with vaccination), the shape of the age profile changes, especially above age 75, and at younger ages, male deaths in 2021 are in fact higher than in 2020. The situation is far more nuanced than could simply arise from the introduction of vaccination in 2021.

Additionally, note that in the first two years of COVID-19, there was no appreciable change in male deaths up to age 37 and up to age 42 in females. For males, there is a slight increase between ages 37 and 42, peaking at age 40 and dropping back to no change at age 42. From age 42 through 64, deaths in 2021 (with vaccination) are higher than in 2020 (without vaccination), while deaths above age 75 in 2021 (with vaccination) are lower than in 2020 (without vaccination). Above age 100, there does not appear to be an appreciable excess of deaths arising from COVID-19, possibly an expression of a particular immune phenotype in the elderly [52,53]. This behavior above age 100 was confirmed in the analysis of the trends in deaths between 2011 and 2021 shown in Supplementary Material S4.

Other year-of-age cohort effects may lie hidden in the data, and for females aged 11–13, deaths are 2.2 standard deviations higher than the expected average in 2021 and 2.3 standard deviations higher for 2020 and 2021 combined. In this same age cohort, male deaths are 2.1 standard deviations lower during 2020 and 2021 combined. A potential effect due to puberty will be discussed later.

### 3.3. Male versus Female Deaths in England and Wales

**Figure 2:**
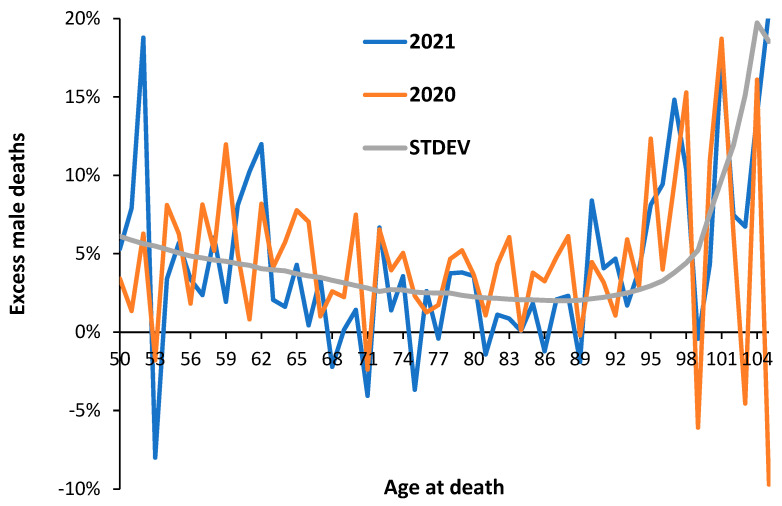
Excess all-cause male deaths (as a percentage difference) during 2020 and 2021 (against the 2019 baseline) across ages 50 to 105+. The gray line shows the value of 1 standard deviation (STDEV) of Poisson variation based on the 2019 baseline.

It is widely recognized that COVID-19 causes higher male mortality [5,6,7,54,55], and the effect against all-cause mortality should be strongly influenced by the higher COVID-19 mortality. This generalization is tested in Figure 2, where the difference between male and female deaths in 2020 and 2021 compared to the 2019 baseline is shown.

From Figure 2, male deaths are generally higher in 2020 (without vaccination) than in 2021 (with vaccination), which tends to suggest that males may have benefitted slightly more from vaccination, although the difference in SARS-CoV-2 variants between the two years may also play a role. Below the age of 50, several ages appear to have significantly greater differences, namely, in 2020, ages 7–9 (males), 12 (females), 33 (females), and 36–38 (females), while in 2021, the ages were 7–9, 12, 11–12, 33, and 36–38, all females. 

Once again, this behavior would be completely obscured using 5- or 10-year age bands. The differences look to be mostly systematic rather than random, and hence, immune/infectious history since birth may be playing a role.

**Figure A2:**
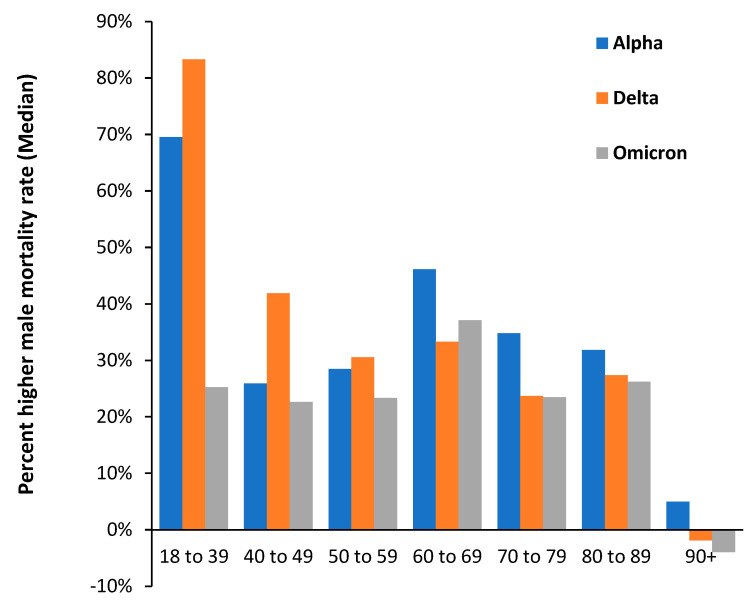
Higher male all-cause mortality rate percentage during SARS-CoV-2 variant circulation for Alpha (January to June 2021), Delta (July to December 2021), and Omicron (January to May 2022) infections in persons who are unvaccinated. Analysis of the data from [33]. The higher male mortality percentage was calculated for each month/age range, and the *Y*-axis shows the median value for each period. All mortality rates are age-standardized within each age band.

The possibility that the peaks and troughs in Figure 2 are systematic rather than random is explored in Figure A2, where the difference in the mortality rate among the unvaccinated (deaths per 100,000 person years) for various age bands is explored for the three variants Alpha, Delta, and Omicron. This data comes from an ONS study relating to vaccination status [34]. In Figure A2, it is apparent that the difference between males and females is highest in the youngest age band of 18–39 years and lowest in the age 90+ group. Up to age 59, the gap between males and females is greatest for the Delta variant; however, above this age, it is highest for the Alpha variant. Omicron has the lowest gender gap except for the age band 60–69, while for age 90+, the gender gap during Omicron reverses to female deaths being higher than males. We are clearly dealing with a complex system where age, gender, and variants interact. Judicious use of both single-year-of-age and age-banded data is required to reveal the full complexity. It is possible that year-of-birth cohorts may lie behind some of the systematic differences.

Given the time-based cumulative effects of various global and individual influences on immunity (specific patterns and timing of pathogen exposure, vaccination with various types of vaccines, sex- and age-related hormonal patterns, distribution of numerous pollutants in the form of pesticides, veterinary antibiotics, radiation, and food additives, among others), there is no reason that males and females of all ages should be equally affected [56]. For example, individuals born after WWI (now aged up to 100 years) had to survive without antibiotics or most modern vaccines until the widespread introduction of penicillin during WWII, with those born after WWII largely benefiting from them. The situation is more nuanced than it first appears, and year-of-age specificity may occur, as has been noted for influenza [56].

### 3.4. Net ‘Real World’ Change in All-Cause Deaths by Age and Gender

**Figure 3:**
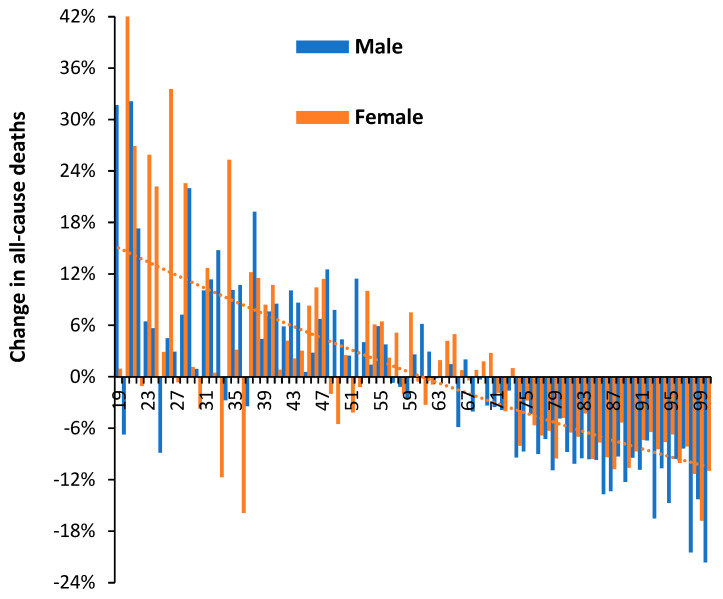
Change in all-cause mortality for males and females during 2021 (with vaccination) versus 2020 (without vaccination). The difference is expressed as a percentage relative to the 2019 baseline.

Figure 3 investigates the ‘real world’ change in all-cause mortality during 2021 (with vaccination + Alpha/Delta variants) compared with 2020 (without vaccination + mainly the original Wuhan strain and some Alpha at the very end of 2020). The term ‘real world’ is used as opposed to the estimated excess mortality either ‘with’ or supposedly ‘due to’ COVID-19. Persons below the age of 19 were generally not vaccinated during 2021—hence the age cutoff in Figure 3. 

Above age 65, males look to have benefited slightly more than females (as shown in Figure 2). Above age 70, all changes show high significance (due to high numbers), while below age 70, changes can be obscured by Poisson randomness. However, the overall trend, which is illustrated by the dotted orange line for females, is clearly toward higher deaths at younger ages. This is also clearly seen in Figure 1. While the lower deaths in 2021 above age 65 may be due to vaccination, it is difficult to attribute vaccination as the sole cause of the generally higher overall deaths below age 65; therefore, more complex issues are involved.

### 3.5. The Delta Variant, Second Half of 2021, Targeted at Younger Ages

**Figure 4:**
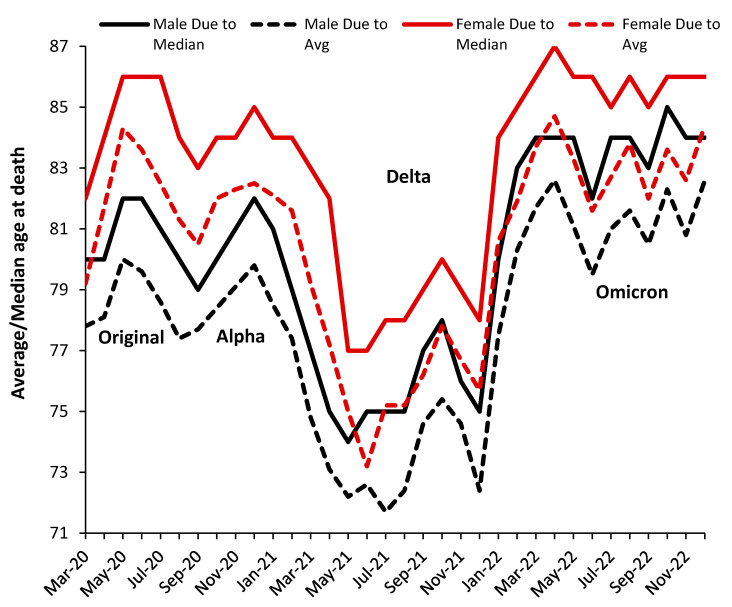
Average/median age at death for males and females in England and Wales for deaths ‘due to’ COVID-19 between March 2020 and December 2022. Data are from the ONS [29,30].

Any COVID-19 analysis will be complicated by the changes in variants over time. We are not aware of any studies looking at the detailed age profile of deaths for COVID-19 variants; however, Figure 4 summarizes the average/median age at death for males and females assessed as ‘due to’ COVID-19. 

In Figure 4, the median is always higher than the average, indicating a skewed distribution. Males die at a lower median and average age. The gap between both average and median age at death for males versus females is around 4 years during the original and the Alpha variants, around 3 years during Delta, and around 2 years during Omicron. Females have a slightly higher gap of 0.4 years (average) between the median and average. The average age at death may slightly increase at the peak of an outbreak, possibly due to additional transmission into care and nursing homes.

Given that the Delta variant originated in India, with a majority younger population, it is unsurprising that this variant was associated with a shift to COVID-19 deaths at younger ages. The seemingly average age at death follows profiles that are seasonal (summer minimum), strain, and outbreak magnitude (peak outbreak maxima) dependent. Seasonal factors are involved in gene expression [57,58]. Figure 4 also allows the transitions between SARS-CoV-2 variants to be visualized. The transitions last several months as the deaths arising from the former variant are replaced by the newer one.

The abrupt changes in the average and median age in Figure 4 strongly suggest that it is the variant rather than vaccination or changes in clinical protocols or drugs that are the primary regulators of the age profile. Vaccination likely modifies the magnitude of the effect of each variant rather than the age profile per se.

**Figure 5:**
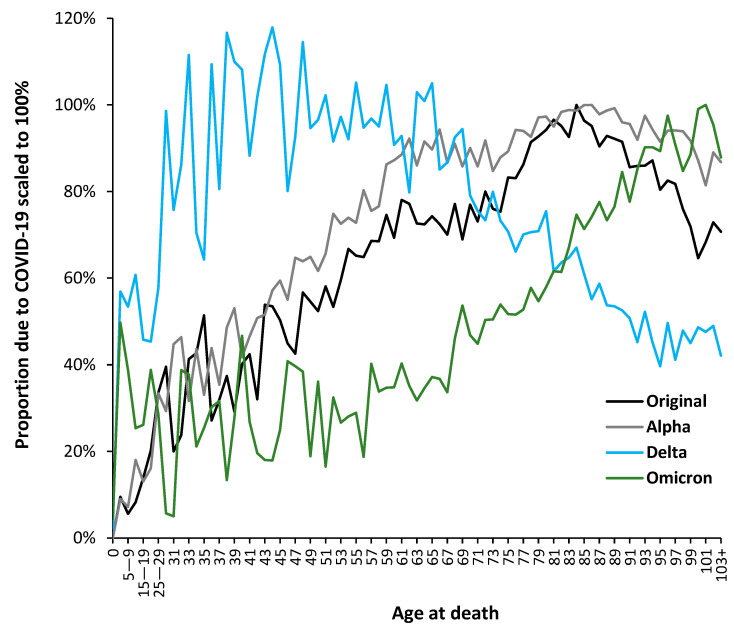
Single-year-of-age (SYOA) profiles for the proportion of total deaths assessed as ‘due to’ COVID-19 in England and Wales. Below age 25, data have been aggregated to ages 1–4, 5–9, 10–14, 15–19, and 20–24. Analysis of raw data in [30].

The above suggests that variant-specific analysis is required. While changes in the average in Figure 4 highly suggest changes in the underlying age profile, the detail can be explored using the proportion of total deaths at each age that are assessed as ’due to’ COVID-19. To this end, Figure 5 presents a detailed analysis in which the trends have been normalized such that they all have the same nominal maximum value.

The results presented in Figure 5 come from a process of evaluating the age profile of COVID-19 deaths in each month from March 2020 to October 2022. This analysis is required because there is a lag between the arrival of the variant, subsequent spread, and then an infectious outbreak followed by deaths. In general, the age profile shows mixed features during a 2-month transition period (see Table 1). The lines in Figure 5 are therefore restricted to data in the non-transition months, which is summed and turned into an overall proportion. Hence, March 2022 to October 2022 for Omicron, etc. The term ‘variant arrives’ indicates the point at which infections commence to the point of being an outbreak. 

In Figure 5, the trends have been scaled to the age at the maximum proportion observed for ages above 40 years. Due to variability in the Delta data, this was scaled to the average age of 33 to 45, which represented the range for maximum deaths. Scaling factors to give 100% for the maximum are: 5.3 for the original strain without vaccine; 4.6 for Alpha; 11.1 for Delta; and 17.8 for Omicron. The higher the scaling factor, the lower the overall mortality. Due to limited data in some of the younger ages, above age 0 and below age 25, data are grouped by 5-year age bands. While there was a succession of variants when Omicron was predominant, there was no evidence for a fundamental change in the age profile.

Further details regarding the relative impact of each variant upon the all-age proportion of total deaths are given in Figure A4 (Appendix A), along with an indication of the time-dependent spread of SARS-CoV-2 variants across the UK, where London is usually worst affected, and that the Omicron variant affected London before the rest of the UK. Hence the need for a two-month window between each variant in Table 1, which also allows for the spread between the regions of England and Wales.

Figure A4 likewise shows that the Delta variant marked a transition to endemic behavior for deaths, which also continued with the Omicron variant. 

The most important point is the divergent age profile for the Delta variant. The population of India contains a far higher proportion of younger people than that of an aging UK population. Unsurprisingly, Delta is optimized for younger ages, with the highest values of death between ages 30 and 65. It also has the highest proportion of deaths among infants under the age of 1 year. The Delta profile (August 21 to December 21) is modified by month-of-year factors and shows a higher proportion of age 65+ deaths compared to the Omicron variant months (March 22 to October 22).

At the other extreme, the age profile for the lower mortality Omicron variant (which seemingly originated in South Africa) has a broad, slightly elevated lower proportion below age 55 and then peaks around age 100—the highest for any variant. The diversity of year-of-age profiles between variants supports the claim that the use of wide age bands is not helpful when attempting to compare results between variants. These findings also concur with our previous age-specific analysis showing high age variability for various influenza outbreaks [56]. Issues regarding the age profile of vaccine effectiveness will be covered in the discussion section. The result is that the period of any study (in different countries/regions) has a material effect on its apparent outcomes.

### 3.6. Modification of the Age Profile in Different Countries/States/Territories

**Figure 6:**
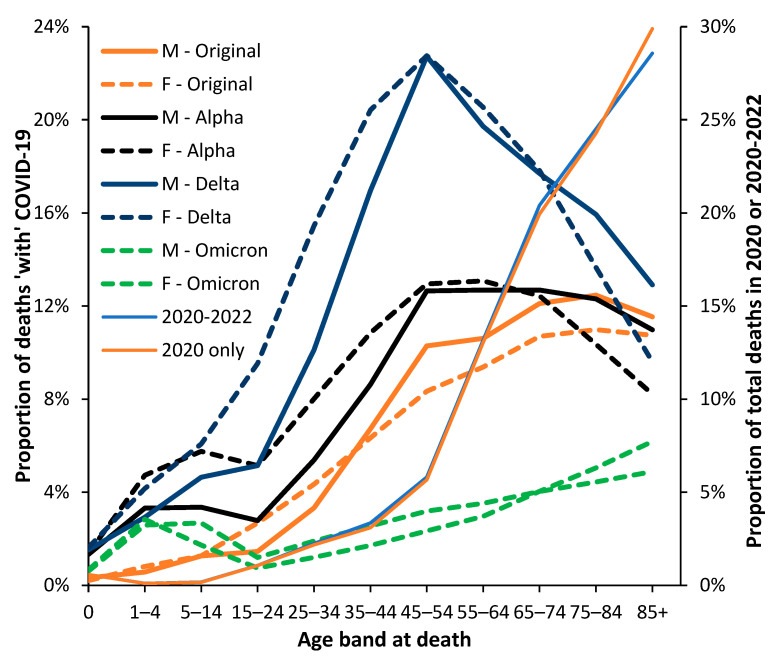
Proportion of male or female total all-cause deaths ‘with’ COVID-19 during each SARS-CoV-2 variant for the USA, as well as the proportion of total all-cause deaths present in each age band. Analysis is from the data in [37].

Each country/state/area has its own unique mix of risk factors for COVID-19-related deaths at each age, such as the proportion of the population in high-exposure occupations, those with obesity or other clinical risk factors, including genetic factors, the risk of transmission due to city/rural living, or risk-taking behavior, etc. [19,20,21,47,48,49,50,59,60,61,62,63,64,65,66,67,68,69,70,71]. This is illustrated in Figure 6, which uses data from the USA rather than England and Wales and includes both males and females. Note that the data from the USA is for deaths ‘with’ rather than ‘due to’ COVID-19 and that only 10-year-wide age-banded data is available above age 1–4 [37]. The red and purple lines give the proportion of total all-cause deaths that occur in each age band. The effect of vaccination on the age profile is addressed in Section 3.8.

As seen from Figure 6, the age profile for the USA is slightly different from that in England and Wales in Figure 5, although the differences are magnified using 10-year age bands in the US data with 85+ as the final age band. This upper-age truncation pushes the profile to the left. The US data has not been scaled as in Figure 5. By way of comparison, Figure 6 shows the proportion of total deaths ‘with’ COVID-19 for both males and females. Recall that the profile of all-cause deaths in males and females is very different, as illustrated in Figure 1, and that cause of death has different profiles between the two genders. However, Figure 6 powerfully illustrates that each COVID-19 variant has an age-specific profile unique to each gender, as was also observed in Figure 2 and Figure 3 for England and Wales. Note that the impact on total deaths (right hand axis) is not greatly shifted by the Delta or other variants since age 45–54 only accounts for 6% of total deaths. The general observation of higher male deaths for COVID-19 is age-, sex-, and variant-specific. A single-year-of-age investigation would likely yield greater detail, as shown in Figure 1.

Hence, despite skewing from the use of age bands in the USA, the shape of the profile is broadly similar to that in England and Wales for all four variants, suggesting that the primary year-of-age profiles are set by the variant and then modified by location-specific factors.

It must be highlighted that the evident transition to childhood COVID-19 mortality for Delta and Omicron seen in Figure 5 and Figure 6 is a strong argument for single-year-of-age studies. The age bands in Figure 6 are likely concealing such behavior. The precise year-of-age detail is required to inform which public health interventions should be favored, such as acquired immunity versus vaccination. Given the very limited number of childhood deaths beyond the first year of life, aggregated data from multiple countries is required. Figure 5 reverted to age bands in childhood because England and Wales had too few deaths to investigate in more detail.

**Figure 7:**
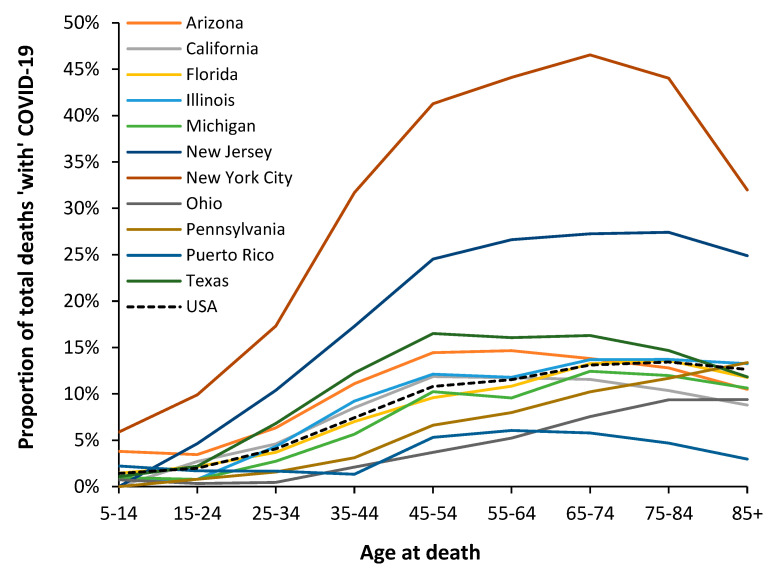
Proportion of total deaths ‘with’ COVID-19 for several American states/territories and for the USA during the period of the original Wuhan strain. Analysis of data from [37]. As mentioned in Section 2.1, the CDC masks death values between 1 and 9 at the state level, and these missing values were interpolated. This mainly affects the two youngest age bands and the smallest states/territories.

Given that the USA is so large, each of the states could effectively be considered a different country. Hence, analysis at the state level is warranted [71]. This is illustrated in Figure 7 using data from American states and the age profile for the original Wuhan strain, which predominated for most of 2020. Note that the proportion of total deaths ‘with’ COVID-19 has not been age-standardized between US states/territories, which will lead to some modification of the profile between states.

Figure 7 shows the magnitude of the Wuhan strain outbreak(s) in each state, with New York having the highest magnitude of impact, followed by New Jersey, and a far smaller impact in Puerto Rico (which has been a US territory since 1898). States such as Alaska and Hawaii had even lower-magnitude Wuhan strain outbreaks, but the data is not shown. Due to the sheer size of the USA, an initial outbreak at the county level took at least 12 months to spread throughout the country, and even after 12 months, some counties had not yet registered a COVID-19 death [71].

**Figure A3:**
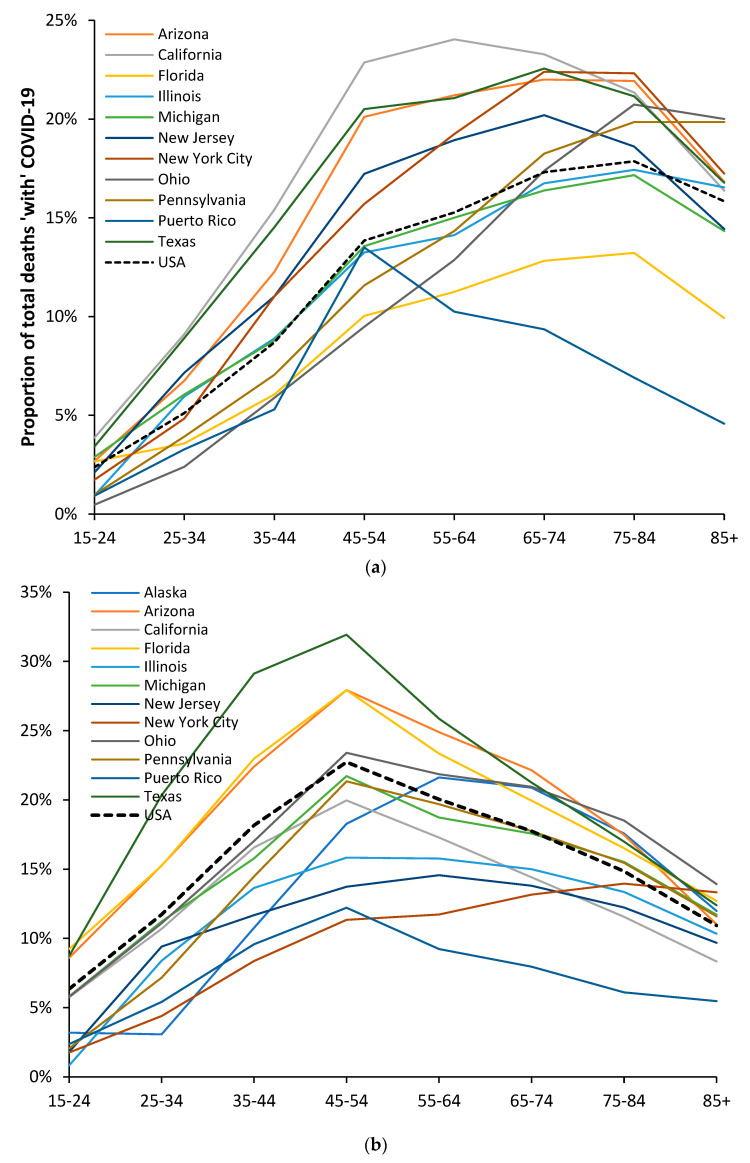

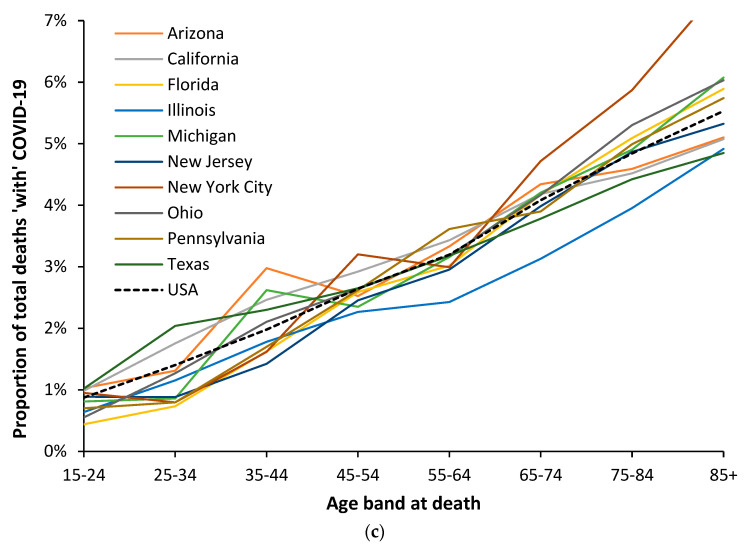
(**a**) Proportion of total deaths ‘with’ COVID-19 in a selection of American states during the period of the Alpha variant. Analysis of the data from [35]. (**b**) Proportion of total deaths ‘with’ COVID-19 in a selection of American states during the period of the Delta variant. Data are from [35]. (**c**) Proportion of total deaths ‘with’ COVID-19 in a selection of American states during the period of the Omicron variant. Analysis of the data from [35]. Lower deaths during Omicron imply that the data below age 55 show higher scatter, especially for the smaller states.

Figure 7 also indicates the slightly modified age profiles depending on state population risk characteristics. Charts showing the shape of the age profile for the Alpha, Delta, and Omicron variants are given in Appendix A, Figure A3a–c.

In Figure A3a, Puerto Rico has a divergent profile from other states/territories for the Alpha variant, while in Figure A3b, the Delta variant peaks in the younger ages, 35–44 in Hawaii, and 45–54 in the other states. In Figure A3c, for Omicron, all states show increasing proportions of total deaths up to age 85+. This upper age band acts to hide the single-year-of-age behavior revealed in Figure 5.

The shape has been subtly modified in each state due to the age-band population risk characteristics of that state and possibly by differential spread through various communities within the state, i.e., popular retirement locations, cities, farming areas, etc. [71]. 

Hence, the overall conclusion remains that each strain has a unique age profile, which is then modified by the mix of population risk factors present in each location at each age.

### 3.7. The All-Cause Mortality Peak for Each Variant

**Figure 8:**
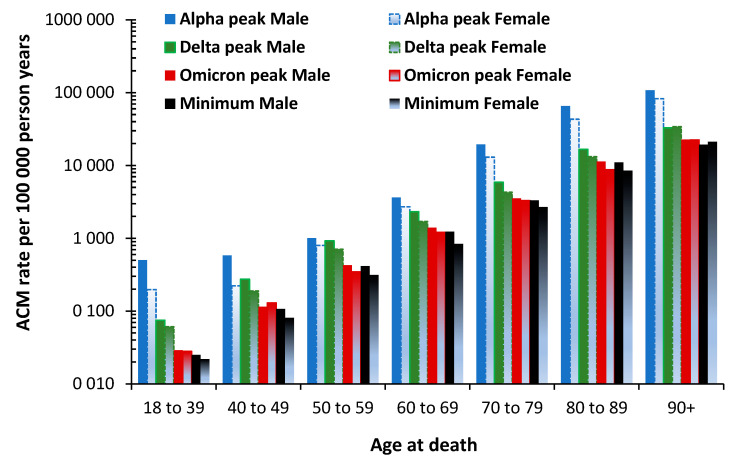
Maximum all-cause mortality (ACM) rate for unvaccinated males and females for the three COVID-19 outbreaks, Alpha, Delta, and Omicron, in England. Analysis of the data in [35].

While Figure 5, Figure 6 and Figure 7 represent a proportion of total all-cause deaths averaged over the period of each variant, the issue of the maximum value of all-cause mortality during the period of each variant modifies the overall number of deaths at each age. To this end, Figure 8 shows the maximum all-cause mortality rate for various age bands in the unvaccinated at the point of maximum monthly mortality rate during the Alpha, Delta, and Omicron variant outbreaks. The Omicron outbreak mostly occurred during the summer of 2022. The minimum all-cause mortality is the lowest value in the time series. The data in Figure 8 come from an Office for National Statistics study regarding vaccination status [35].

Note that the *Y*-axis is a logarithmic scale. Starting from around 100,000 all-cause deaths per 100,000 person years at ages 90+, the mortality rate rapidly drops below 1000 deaths per 100,000 person years—a 99% reduction—for ages below 60 years.

The shape of the profile within each age band varies considerably. For example, a moderately even reduction across variants for the age band 60–69 compared to a rapid decline across the original and Alpha variants at age 18–39, etc. Likewise, the gap between males and females varies by variant and age band, as noted in Section 3.3.

Also note that by 2022, during an outbreak of one of the high transmission-low mortality Omicron variants, the all-cause mortality rate was substantially reduced, especially in the age band 18–39. The difference in the mortality gap between males and females reduces with each successive variant. The minimum value represents the baseline all-cause mortality rate. The Omicron outbreak is barely above the minimum.

These results confirm the profiles for age, gender, and variant seen in the USA in Figure 6.

### 3.8. The Age Profile of the Unvaccinated versus the Vaccinated

**Figure 9:**
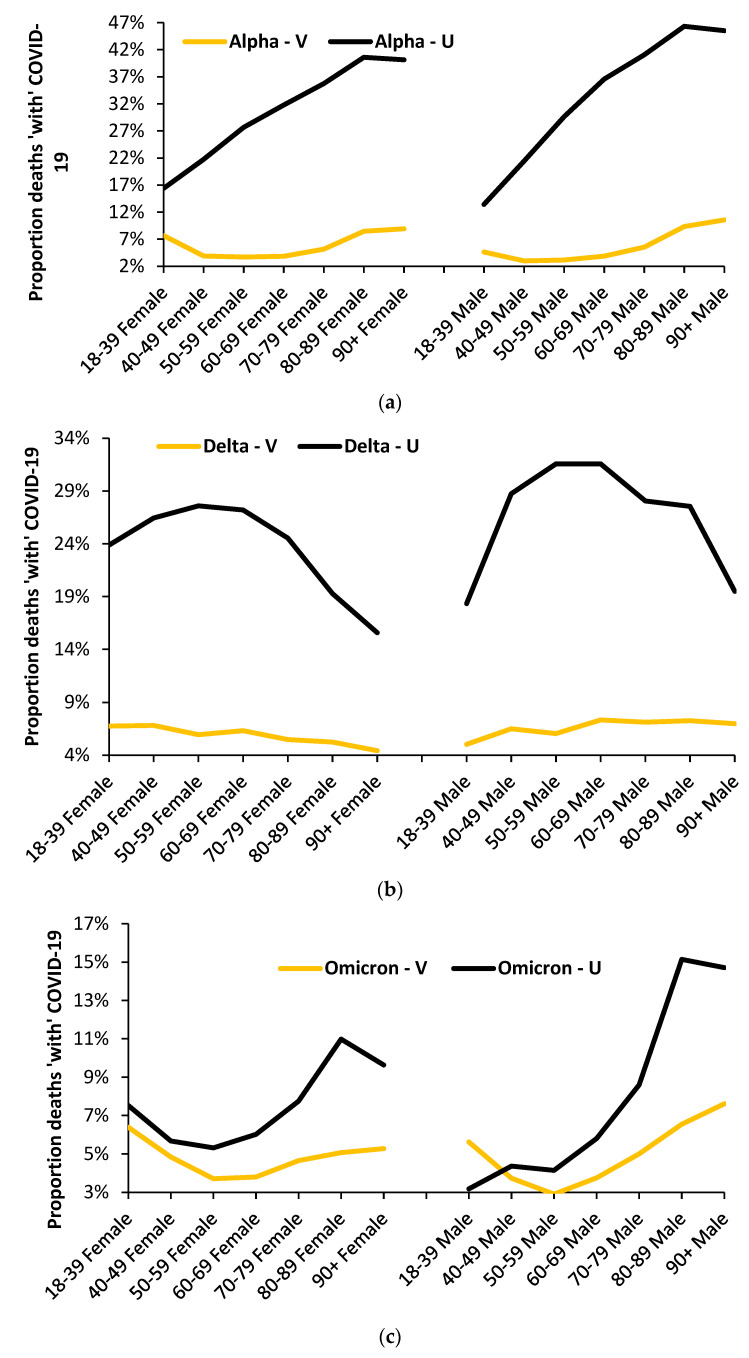
(**a**) Effect of vaccination upon the shape of the age-banded profile in England for male and female deaths during the Alpha variant outbreak. Analysis of the data in [35]. Data for ‘vaccinated’ includes persons with one or more doses of vaccine plus a vaccination status group in the first 21 days after vaccination [35]. V = vaccinated, U = unvaccinated. (**b**) Effect of vaccination upon the shape of the age-banded profile for male and female deaths during the Delta variant outbreak in England. Analysis of the data in [35]. (**c**) Effect of vaccination upon the shape of the age-banded profile for male and female deaths during the Omicron variant outbreak in England. Analysis of the data in [35].

Figure 6 suggests that various factors, including vaccination, may combine to alter the shape of the age profile, and so Figure 9a–c investigates whether vaccination makes a unique contribution.

The data in these figures come from the Office for National Statistics study on deaths during the COVID-19 pandemic by sex, age band, and vaccination status [35]. As such, it has certain limitations in that there is no data below the age of 18, there are only 7 age bands, and though the data is summed over time for each variant, the outcome is not age-standardized within each age band. However, the data is sufficient to illustrate the point that each variant has a unique age profile in both the unvaccinated (U) and the vaccinated (V). Lastly, in the UK, a mix of different vaccines was employed over time. The UK government does not publish data on the mix of vaccines, and the results for the vaccinated group cannot be attributed to any vaccine type [35].

From Figure 9a, it is evident that males/females have slightly different age profiles, as demonstrated previously in this study. The next point is that most deaths occur in the unvaccinated, and this means that the age profiles shown earlier in this study are not greatly affected by using all deaths ’with’ COVID-19.

Hence, during the Alpha outbreak, the shape of the age profile was roughly consistent between the vaccinated and unvaccinated, except for the vaccinated 18–39 age band, which experienced increased deaths (especially females) relative to other vaccinated age groups.

The situation for Delta (Figure 9b) in the unvaccinated is consistent with previous figures for Delta, with the maximum proportion of COVID-19 mortality around age 50–59. For the unvaccinated, there are divergent age profiles compared to the vaccinated, though the deaths are not age-standardized within each age band. However, the profile of the vaccinated is confirmed in another study [72]. Once again, lower deaths among the vaccinated do not unduly influence the overall outcome.

The situation for Omicron (Figure 9c) in the unvaccinated is consistent with previous figures, except perhaps for those aged 90+. On this occasion, recall that age standardization within the 90+ age band would change the result, as per Figure 2. Note the strong difference between males and females for age bands below 60 years. However, the profile for the vaccinated is confirmed in another study [72], where the vaccinated do indeed fare worse for males aged 18–39—although this needs to be qualified to a single-year-of-age context.

In conclusion, fewer deaths among the vaccinated implies that a combined age profile that includes both the vaccinated and unvaccinated does not unduly affect the outcomes in the previous figures. Given the differences between male and female outcomes, all analysis must be split by sex. Finally, the population-wide impact needs to be understood in a single-year-of-age context, as in Figure 2, illustrating our argument that the use of age bands can obscure the underlying reality.

## 4. Discussion

### 4.1. Impact of SARS-CoV-2 Variants on Disease Severity and Mortality

The year-of-age profiles in this study confirm a trend toward reducing mortality with the progression of variants. This is especially so for the Omicron variant [5,6,7]. For example, a study by the ONS in England regarding the mortality from Omicron versus Delta showed an 87% reduction at ages 15–59, an 86% reduction at ages 60–69, and a 55% reduction at ages 70+. The reduction in mortality was gender-specific, with a 75% reduction in males but only a 56% reduction in females [64]. Such differential effects on males/females are confirmed in this study.

These results are broadly consistent with the pattern of all-cause mortality in Figure 5 and Figure A2, where a shift to younger ages is highlighted for the Delta variant. However, the year-of-age profiles in Figure 5 provide far greater detail, especially below age 55. The elevated mortality for Omicron in the elderly, especially beyond age 70 and peaking at age 100/101, shown in Figure 5, is obscured in most other studies due to the use of age 85+ or 90+ end points.

### 4.2. Local Risk Factors

At the local level, the unique age profile for each SARS-CoV-2 variant is likely to be the combined effect of a variety of mechanisms, namely:

1. Age profiles for infection by the virus due to social networks or low risk aversion, i.e., nightclub and other large event attendance [65].
2. Proportion of persons in each group in high-exposure work categories, i.e., healthcare staff, taxi drivers, teachers, police, bar and restaurant staff, etc. [66].
3. Family transmission risk factors such as household crowding [67].
4. Proportion of persons in each age group with a high risk of mortality due to clinical risk factors, i.e., obesity, multiple morbidities [68], activated genetic risk factors (as opposed to epigenetically silenced genetic risk factors) [69,70]—including ACE 2 receptor mutations [71].
5. Level of vaccination and the mix of vaccines employed.

Hence, the observed age profile should vary by country and region as the interactions between the above factors modify the actual year-of-age mortality. This was illustrated in Figure 7 and Figure A3a–c using data for American states, where the height of the line represents the magnitude and the shape of the line illustrates the location-specific modification due to the #4 point above.

The fact that the mix of risk factors can vary by year-of-age has been largely overlooked and almost rarely measured at that degree of detail.

### 4.3. The Shift in Mortality to Younger Children Subsequent to the Wuhan Strain

We suggest that as a result of the first year of circulation in the human population and because of the virus’s adaptation to a new host (the human), the virus’s receptor-binding sites changed and increased their affinity (tropism) to various human receptors (cell surface proteins), including increased affinity to children’s cell receptors. The virus replication machinery may also have adapted to some peculiarities in children’s cell metabolism and gene expression patterns. This could explain why, in 2020, there was no mortality in children 0–10 years old (because their cells were not permissive for virus adhesion and replication) [30].

However, the high natural changeability of the virus plus the vaccination-accelerated changeability resulted in the adaptation of some virus subpopulations to replication in children’s cells. In England and Wales, the first ‘due to’ COVID-19 deaths in infants under 1 did not occur until August 2021, October for age 1, November for age 2, and December for age 3, which was the beginning of the Delta variant outbreak [30]. Age 5 deaths ’due to’ COVID-19 did not occur until May 2022 [30], i.e., the month of death increases in order of increasing age.

### 4.4. Year-of-Birth Cohort Effects

Throughout the study, we have hinted at the existence of year-of-birth cohort effects (Figure 1, Figure 2 and Figure 3, etc.), which lead to specific ages (or, more correctly, years of birth) where there is a clustering of high/low behavior. We went to some lengths in Supplementary Materials S2–S4 to establish a reliable year-of-age profile for 2019 (the baseline before COVID-19) to give some assurance that such birth cohorts were real. Some variation in apparent magnitude will occur due to Poisson variation, as per Figure A1. Birth cohorts will arise from the exposure of infants to a variety of pathogens plus additional environmental effects [27]. Year-of-birth cohort effects are a well-recognized phenomenon in disease expression [73,74,75,76], and it would be extremely surprising if they were absent during the COVID-19 pandemic. 

As far as we are aware, there are no other studies investigating birth cohort effects upon infection with, or the severity of, COVID-19 disease. Once again, such studies can only be conducted with year-of-age data.

The identification of birth cohorts is important for two reasons:

1. Which birth cohorts show enhanced resistance to COVID-19 infection and death?
2. Which birth cohorts show an enhanced response to COVID-19 vaccinations?

We point out that the WWI birth cohort either survived the 1918–1919 Spanish flu pandemic or was born to parents who had survived the pandemic. Our analysis shows that this cohort seemed to experience little, if any, increase in deaths during the COVID-19 pandemic. Genetic material retained in biobanks may shed light on the issue. Further studies on the topic of birth cohort effects are highly recommended.

### 4.5. Puberty and Excess Female Deaths

On the other hand, some infections and conditions are specifically age-related [77,78]. For example, the Japanese surveillance system for infectious diseases was used in the analysis of seven viral and four bacterial infectious diseases [77]. The male-to-female morbidity (MFM) ratios in different age groups were estimated. MFM ratios were > 1 for five viral infections out of seven in childhood, i.e., male children were more frequently reported as infected than females with pharyngoconjunctival fever, herpangina, hand-foot-and-mouth disease, mumps, and varicella. More males were also reported to be infected with erythema infectiosum (parvovirus B19) and exanthema subitum (roseola), but only in children under 1 year of age. By contrast, in adulthood, the MFM ratios decreased to < 1 for all the viral infections above except varicella, i.e., adult women were more frequently reported to be infected than men. Sex- and age-related differences in reported morbidity were also documented for bacterial infections. Reported morbidity for enterohemorrhagic *Escherichia coli* infection was higher in adult females, and females were reportedly more infected with mycoplasma pneumonia than males in all age groups up to 70 years old [77]. Such factors will lie behind the age–gender differences seen in this study.

Another systematic analysis identified 142 datasets with information on severity of disease by age for 32 different infectious diseases: 19 viral and 13 bacterial [78]. It was commonly seen that for almost all infections, school-age children suffered the least severe diseases, and severity starts to rise long before old age. For many infections, even young adults suffer more severe diseases than children, and dengue was the only infection that was most severe in school-age children. Together with data on vaccine response in children and young adults, the findings suggest peak immune function is reached around 5–14 years of age. Relative immune senescence may begin much earlier than assumed before accelerating in older age groups. They suggest implications for understanding resilience to infection, optimal vaccine scheduling, and appropriate health protection policies across the life course [78]. 

A cluster of excess female all-cause deaths aged 10–13 was noted in Section 3.2. This was matched by a deficit in male deaths at this age, which suggests a common link to puberty. During puberty, females develop heightened immune reactogenicity [79,80,81,82], which is accompanied by a very high number of epigenetic changes (seven times more than boys) in mainly high-affinity estrogen response genes involved in immune and inflammatory functions [78]. Similar hormonal effects occur during puberty in males [79,80,81,82]. On this occasion, such hormonal effects during puberty appeared to protect adolescent males but disadvantage females.

This leads to the interesting possibility that age-matched females for pre-, during-, and post-menopause may experience modified age profiles and/or different mortality rates ‘due to’ COVID-19.

We infer that human year-of-age susceptibility to pathogens outlined above must interact with the year-of-age profiles for death of the COVID-19 variants and other pathogens. Such interactions are implied in a recent study relating to pathogen interference [56] and will now be discussed in greater detail.

### 4.6. Structure–Function and Other Aspects of Variant and Age Dependence

In this section, we present a framework to interpret how various factors interact to determine the final age profile and then examine several important aspects within this framework.

SARS-CoV-2 variants arise from the well-recognized virus mutation process [8,9,10,11,12,83,84,85]. Millions of random mutations leading to new variants are thrust out into the world’s human population and its various people groups. Most mutations fail to thrive, but those that do can further ‘optimize’ their genetic make-up in a manner specific to the people group and/or environment where they have become entrenched. Such genetic make-up may extend beyond that regulating the surface of the virus, although wider genetic aspects are poorly studied [85].

Virus infectious process initiation and outcomes depend on multiple conditions:

1. Biological properties and peculiarities of the virus species.
2. Biological properties and peculiarities of a potential host.
  - Strength of innate and adaptive immunity against specific pathogens
  - Presence and distribution (localization) of appropriate cell receptors for the initial adsorption of virus particles
  - Presence and activity of cellular components necessary for virus’ cell entry and virus genome release, i.e., function of pinocytosis, the presence and activity of specific proteolytic enzymes in the cytoplasm, etc.
  - Presence and activity of specific cellular RNA- or DNA-replication pathways
3. Environmental conditions.

Therefore, to find reasons for the age specificity of an infection, i.e., conditions when #1 and #3 are compatible, we should try to find age-related differences in #2 by studying age-related differences in immunity, protein composition, and the RNA/DNA replication machinery of host organisms. For this purpose, it is worthwhile to search for age-related gene expression patterns (peculiarities) in the host organism and to consider possible interrelationships between such differences and virus replication activity. 

There are numerous research studies demonstrating age-related differences in gene expression of various cellular components, which can be responsible for age-related variations in virus adsorption, entry, virus RNA release, virus RNA replication, offspring virus particle assembly, and shedding [86,87]. Certainly, there are gene expression-related reasons for age-related variations in immunity [88].

For example, significant changes in the immune system during puberty arise due to hormonal rearrangement [78,79,80,81]—as discussed above. This may cause enhanced susceptibility to some infections in children in puberty and explain age- and sex-related differences in disease incidence within the population [77,78].

In modern conditions of active, massive anthropogenic biological interventions (multiple vaccinations, changes in food components, etc.), there are potential reasons for the age-specificity of some infections. For example, extensive vaccinations with various vaccines for specific age groups may provide them with increased protection against the same or different pathogens—as observed in previous BCG vaccination studies on susceptibility to COVID-19 infection and mortality [89,90].

The situation regarding #1 and how specific mutations can confer age-specificity depends on both #2 and #3. However, this is a vastly neglected area of study, and several possibilities will now be discussed.

#### 4.6.1. Roles for Immune Priming in Age Specificity

Both influenza and α and β coronaviruses, of which the α types are HCoV-NL63 and HCoV-229E, while the β are HCoV-OC43 and HCoV-HKU1, have been infecting humans for many thousands of years [91]. However, SARS-CoV-2 is sufficiently different from previous coronaviruses to create a unique set of immune issues that can be illustrated by influenza.

In the case of influenza, the age specificity of different clades (variants) is greatly influenced by the phenomenon of ‘antigenic original sin’ [92,93,94], or more recently called ‘antigenic priming’ [94]. The previous (usually in childhood—called a birth cohort) exposure to one clade will generally invoke a response to the first clade when exposed to an antigenically different one. This response magnifies the disease in that birth cohort because the ‘wrong’ antibody is produced. However, when the new clade is sufficiently similar to the former clade, the response is helpful because both B- and T-cells have already been trained, and the time taken in the training process can be avoided, resulting in a faster immune response [93,94,95]. On this occasion, natural immunity can be more effective than vaccination, which can be unhelpful if the antigenic mix in the vaccine is dissimilar to the circulating strains.

This process was amply demonstrated during the 2009 Swine flu (H1N1) pandemic, where persons aged over 65, i.e., born before 1944, had been exposed to an antigenically similar clade during the 1918–1919 Spanish flu pandemic, which continued to circulate through to 1957 [96,97,98,99]. In the USA, some 65% of those over 65 had existing immunity [96]. On this occasion, age 65+ is a commonly used age band in influenza vaccine effectiveness studies, and hence, based on the 1957 end point for the post-1918 pandemic H1N1 variants, persons aged 52+ would have pre-existing immunity but probably not above age 91+. Therefore, mortality was skewed toward the younger age groups, who had no previous exposure [96,97,98,99]. However, severity was seemingly higher in the Americas than in Australia, New Zealand, and Europe [95,96], possibly due to regional differences in the prevalence of the 1A.1.1.3 and 1B.2.1 genetic clades [100].

As noted earlier, a skew to younger ages occurred in the 1918–1919 Spanish flu pandemic because 28-year-olds had been previously exposed as infants to the antigenically dissimilar 1889–1890 Russian flu pandemic [27]. The Russian flu had a unique timeline of international spread [101], and so an exact age of 28 will slightly vary between countries.

The situation is slightly more complex since repeat influenza infections/vaccinations leave their own antibody landscapes [102], although childhood exposure remains a dominant force [103]. Hence, immune priming is part of far wider antigenic landscapes that develop over time from repeated infection with variants of the same virus. Vaccination seemingly activates antibodies to all previous variants encountered by the individual, irrespective of antigenic distance, although the primary strain encountered in early life shows the highest antibody response [103]. Antigenic priming is a more nuanced and ongoing process.

In the case of coronaviruses, none had made the huge antigenic leap that occurred in SARS-CoV-2. Among the coronaviruses, HCoV-NL63 and HCoV-OC43 infections are common in infancy and look to confer some protection against subsequent HCoV-229E and HCoV-HKU1 infections [91]. 

Such birth cohorts were first observed in influenza [92], but they apply to any virus that has different strains and variants [93,94,95]. Hence, exposure of infants to antigenically similar strains of other α and β coronaviruses—most likely those causing the common cold—may account for limited birth cohort effects [91]. Such an effect seems to apply to SARS-CoV-2 infection, but in a more limited way [96], and to COVID-19 vaccination [93].

It is also highly likely that the process of antigenic priming will begin to affect emerging variants following initial exposure to the Wuhan or subsequent strains. Acquired natural immunity (from any variant) has been shown to confer protection against the subsequent effects of further COVID-19 infection [104,105,106,107,108]. The extent of exposure to each variant is highly dependent on location, as shown in Figure 7 and Figure A3a–c.

Hence, regarding the potential role of antigenic priming upon the year-of-age profiles of SARS-CoV-2 variants, we conclude that this virus has not had a very long-term interaction with the human population as seen in influenza and that such mechanisms are only beginning to play a central role in the expression of the age profile—although in the sense that antigenic priming only acts as yet another factor to modify the intrinsic age profile of the particular (future) variants that would otherwise be expressed in a ‘blank sheet’ population.

#### 4.6.2. Roles for Pathogen Interference

COVID-19 infection does not operate in supreme isolation from other pathogens [56,109,110,111], and the interaction between pathogens through the effects against small noncoding RNAs (miRNAs) may contribute to year-of-age effects—under the likelihood that each SARS-CoV-2 variant can instigate differential patterns of miRNA production [112,113,114,115,116,117,118,119]. In cancer patients, both COVID-19 and other pathogens intersect at NAMPT/NAD metabolism, which can direct both innate immune cell effector functions and homeostatic robustness in both cancer and infection [120].

Previously, we emphasized the role of small noncoding RNAs (miRNAs) in gene expression and the processes of ‘pathogen interference’ [56]. Among a multitude of functions, miRNAs are central to metabolic regulation [112,121]. Cells produce a multitude of miRNAs when infected with COVID-19 [112,113,114], and COVID-19 also has several miRNAs that are coded into its genome [115,116,117,118,119]. 

It is important to note that all major COVID-19 risk factors are associated with dysregulated miRNA profiles, such as obesity [122], diabetes [122,123], heart disease [124], and cancer [120,121,125]. It has been suggested that miRNAs are involved in unusual all-cause mortality outcomes following COVID-19 vaccination [74].

Relatively stable extracellular miRNAs are present in most biological fluids. These circulating miRNAs are transported by membrane-derived vesicles (exosomes and microparticles), lipoproteins, and other ribonucleoprotein complexes [126]. Such miRNAs are selectively exported from cells with distinct signatures that have been found to be altered in many pathophysiologies, as observed above. Functional miRNAs are delivered to recipient cells by various routes, using cellular machinery to reduce target gene expression and alter the cellular phenotype. The miRNAs mediate cell-to-cell communication, linking disparate cell types, diverse biological mechanisms, and homeostatic pathways [126]. Although significant advances have been made, miRNA intercellular communication is full of complexities, and many questions remain unanswered [126]. However, such selective export of miRNAs seems to explain why different studies have detected a different range of miRNAs following COVID-19 infection.

The processes of aging are evident in human miRNA production. For example, miRNA 92a declines with age in CD3 + CD8 + CD62L + cells and CD8+ T-lymphocytes. This suggests that the age-related attrition of human naïve T-cells could be connected to a reduced miRNA-92a level in T-lymphocytes, and downregulation of the miRNA-92a level might indicate exhaustion of naïve T-cells due to alteration of the immunologic condition with aging and hence in vaccine response [127]. How the year-of-age profiles for each variant will modify the range of miRNAs arising from COVID-19 infection remains an unexplored area. Sites specific to miRNA production appear to reside on the X-chromosome [128], which may explain the higher male mortality from COVID-19 and is related to wider genetic polymorphs associated with higher COVID-19 risk [129,130,131].

These factors may interact with the age specificity of SARS-CoV-2 variants in yet unknown ways.

#### 4.6.3. Viral Entry as a Molecular Signal Event

The fact that the SARS-CoV-2 variants have recognizable changes in the spike antigen [132,133], responsible for binding to the cell membrane, provides a clue to the molecular processes behind age specificity. Several potential mechanisms are available, namely hydrogen bonds and hydrophobic interactions between the variants and the ACE-2 receptor sites [132,133,134,135] and previously unexplained variations in the small noncoding RNAs (miRNAs) produced in response to COVID-19 infection, which then alter gene expression.

It has been recognized for many years that the production of miRNAs occurs in response to ‘environmental cues’, leading to systemic signaling [136]. Exactly how this happens has never been elucidated, i.e., miRNAs can be observed to appear in a cell, but there is no knowledge of how that pattern of miRNAs was selected.

Following infection, many viruses produce fusogen proteins, which then lead to further fusion of infected cells with nearby uninfected cells [137,138]. This includes members of the coronavirus family, such as COVID-19. This leads to the formation of giant cells called syncytia, mostly from cell–cell fusion of alveolar epithelial cells and from cell–cell fusion of infected alveolar macrophages [138].

The action of fusogens leads to lipid rearrangements in the membrane [138]. We can assume that viral fusion and entry trigger a host of signaling events that lead to miRNA production. The molecular process called ’pathogen interference’ is one of many observable outcomes of the action of miRNAs [56]. In this instance, the miRNAs then regulate the production of various interferons, which in turn act to limit or enhance infection by a second pathogen [139,140].

We propose that it is the spectrum of miRNAs released following entry into a cell that is the primary basis for the observed age profiles of different SARS-Cov-2 variants.

### 4.7. Personal Risk versus Population Risk

We have previously emphasized the role of genetic risk and the substantial variation in human immunity regarding ‘personal’ risk as opposed to population risk [56]. Several loci for genetic risk due to COVID-19 have likewise been identified [141,142,143,144], and the expression of genetic risk is age-dependent [141,142,143,144]. Likewise, there is no single antibody against COVID-19; rather, humans produce a wide variety of antibodies, some of which are more effective than others [145]. Individuals experiencing sequelae after acute COVID-19 infection experience an aberrant immune response, which is revealed by subsequent vaccination [146]. Once again, it is unknown how such factors interact with the year-of-age profiles for SARS-CoV-2 variants.

### 4.8. Limitations of the Study

The study only covers two high-income countries for variants that were common in England and the USA. Further studies are needed to document the age profiles of the other variants, such as Beta, Gamma, etc., that occurred in various countries.

Two methods were used to attempt to characterize the age profiles. The method based on annual deaths relied on establishing a 2019 baseline. This method showed promise; however, annual totals are not applicable to specific COVID-19 variants. In theory, the method could be modified to cover periods relevant to each variant. Nevertheless, significant shifts in the age profiles were observed.

In the second method, the proportion of total deaths ‘with’ or ‘due to’ COVID-19 for each age was used to construct age profiles for each variant in England and Wales and the USA. In this approach, monthly data is aggregated to cover the period over which each variant operates, and the period of transition from one major variant to the next can be observed. Even though it is impossible to determine which death was clinically ‘due to’ COVID-19, the use of ‘with’ or assessed as ‘due to’ appears to provide an adequate assessment of the age profile. The inability to accurately assess ‘due to’ COVID-19 deaths remains an international problem; however, it should not unduly affect the age profile. As we have pointed out, during a pandemic, speed is of the essence so that the public can be rapidly informed as to which ages have the greatest risk of mortality.

It is unknown how vaccination based on the original strain will affect the age profiles in 2021 and beyond. For a perfect vaccine, the age profile could be expected to shift in proportion to the vaccine coverage for each age. However, the COVID-19 vaccine has far more nuanced effects against all-cause mortality than has been appreciated, especially for different COVID-19 variants [74]. However, vaccination is incapable of explaining the unique age profile for the Omicron variant, where deaths are highest among those ages who are the most vaccinated, i.e., age 90+. This could be resolved by comparing the proportion of total deaths due to COVID-19 in the vaccinated and unvaccinated groups. The observed highly nuanced behavior with age bands [74] once again strongly suggests that single-year-of-age is the gold standard for assessing cause and effect. 

Finally, we have demonstrated that the age profile for each variant should strictly speaking be determined among the unvaccinated, but that fewer deaths among the vaccinated implies that an age profile based on the unvaccinated plus the vaccinated is a useful proxy.

### 4.9. Wider Application

Given the fact that the age profile for COVID-19 deaths has central implications as to which ages should be vaccinated (especially in resource-constrained countries), it is recommended that all countries report the year-of-age proportion of weekly deaths that are either ‘with’ or ‘due to’ COVID-19—especially split by sex. These data can be interim and then updated later. The WHO may be a logical organization to conduct this analysis. In this manner, emerging shifts in the age profile can be identified early rather than later, as speed rather than accuracy is of the essence. The public can then be warned to take appropriate voluntary age-specific measures, and public health agencies can modify vaccination campaigns accordingly.

The circumstances surrounding the Delta outbreak in India showed the opposite approach to our data-sharing recommendation. For whatever reasons, the Indian government appeared intent on denying that there were high death rates during the Delta outbreak, which occurred in early 2021 in India [147,148]. As a result, accurate data collection seemed to be discouraged, and Indian nationals were permitted to travel abroad, thereby spreading this variant and leading to the Delta surge in the second half of 2021 (Table 1) seen in the UK and USA. Had a single year-of-age profile for deaths from the early Indian outbreak been available, hundreds of thousands of world-wide deaths could have been prevented. It is unvaccinated males that seemingly make up the higher deaths below age 70, as seen in Figure 1, and Figure 9b clearly shows that vaccination was highly effective for younger males during the Delta outbreak. Sadly, the opportunity was lost.

It is of interest to note that Griette et al. have developed a mathematical approach to the specific age dependence for infection and death from COVID-19 [149]. Their model, which uses age bands, was applied during the outbreak of the original Wuhan strain in Japan and could easily be adapted to single-year-of-age profiles for both males and females for the new COVID-19 variants and other epidemics and pandemics.

## 5. Conclusions

This study has established that each SARS-CoV-2 variant has an intrinsic year-of-age profile relating to death, which is then modified by population characteristics such as gender, the proportion of persons at each age with high occupational, clinical, or genetic risk, and vaccination status, as demonstrated by differences between US states. 

Similar unique age profiles for death have been reported for influenza clades [56]. We are unaware of any studies that have been directed at the question of whether the difference in the age profile of the variants upon which vaccines are based and the prevailing variants has a major effect on vaccine effectiveness (VE). We note that VE for influenza vaccines can occasionally be negative [56]. This has been assumed to be due to ‘antigenic distance’ between the circulating clade and the administered vaccine [56], which may also include aspects of the differences in age profiles [56]. It is highly likely that birth cohort effects are present during COVID-19-induced mortality. 

**Figure A4:**
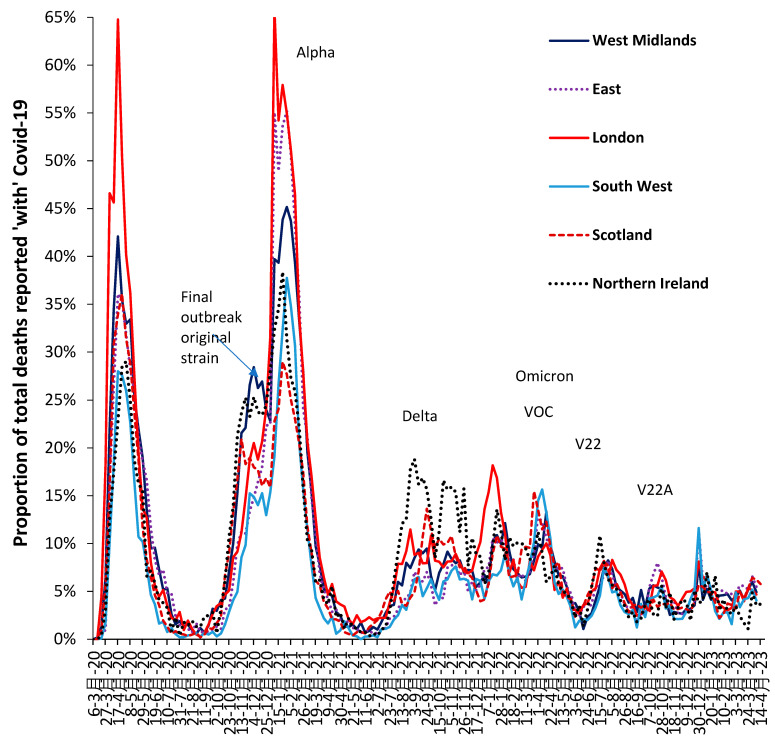
The proportion of all-ages weekly deaths reported as ‘with’ COVID-19 for various parts of the UK, from 6 March 2020 to 14 April 2023. Analysis of the data from [32]. Due to a deficit in COVID-19 testing capacity, the magnitude of the first peak may be underestimated. As per Supplement S5, the real mortality ‘due to’ COVID-19 will be lower than the ‘with’ COVID-19 data suggest.

The high ambiguity in establishing if a death was directly caused by COVID-19 implies that establishing a year-of-age baseline for 2019 is vitally important. We recommend wider studies on the topic of age specificity for COVID-19 variants with data originating from different countries with markedly different population age structures and levels of poverty, along with greater exposure to some of the other variants that were not common in the UK and USA. We have proposed that small noncoding RNAs may regulate the age profiles for COVID-19 variants, and this requires further investigation. Indeed, the sudden transition of COVID-19 outbreaks from seasonal to endemic (as per Figure A4) for both the Delta and Omicron variants requires a detailed examination of potential changes in miRNA profiles using blood stored for research.

## Figures and Tables

**Table 1:** A time profile for months in which deaths are dominated by a single variant in England and Wales. (β)—the absolute magnitude of COVID-19 deaths may have been underestimated in the early months because testing capacity was inadequate [14]. The point of maximum deaths aged 65+ for each variant is shown in bold.

Month	Proportion of Total Deaths Due to COVID-19: Average Age 65+	Variant Arrives (Infections)	Variant Dominates Deaths
October 2022	4%		Omicron
September 2022	2%		Omicron
August 2022	4%		Omicron
July 2022	4%		Omicron
June 2022	2%		Omicron
May 2022	4%		Omicron
**April 2022**	**7%**		**Omicron**
March 2022	5%		Omicron
February 2022	6%		Mixed Delta/Omicron
January 2022	8%	Omicron starts	Mixed Delta/Omicron
December 2021	5%		Delta
**November 2021**	**7%**		**Delta**
October 2021	6%		Delta
September 2021	6%		Delta
August 2021	5%		Delta
July 2021	2%		Mixed Alpha/Delta
June 2021	1%	Delta starts	Mixed Alpha/Delta
May 2021	1%		Alpha
April 2021	2%		Alpha
March 2021	9%		Alpha
February 2021	30%		Alpha
**January 2021**	**38%**		**Alpha**
December 2020	22%		Alpha
November 2020	19%		Alpha
October 2020	7%		Mixed Alpha/Original
September 2020	1%	Alpha starts	Mixed Alpha/Original
August 2020	1%		Original
July 2020	3%		Original
June 2020	9%		Original
May 2020 (β)	23%		Original
**April 2020 (β)**	**32%**		**Original**
March 2020 (β)	3%		Original

## Data Availability

All data are publicly available, and sources are listed in the Materials and Methods section.

## Supplementary Materials

The following supporting information can be downloaded at: https://www.mdpi.com/article/10.3390/idr15050058/s1. Section S1: Actual deaths versus forecast deaths in England (2001–2061); Section S2: Methods for adjusting deaths to the 2019 baseline value; Section S3: Adjustment factors based on births to give a 2019-equivalent estimate; Section S4: Results for the alternative methods to calculate the 2019 ‘average’ number of deaths; Section S5: Deaths assessed as ‘due to’ COVID-19 in 2020 and 2021 compared to excess deaths relative to 2019.
