# Supplementary material for: COVID-19-Related Age Profiles for SARS-CoV-2 Variants in England and Wales and States of the USA (2020 to 2022): Impact on All-Cause Mortality
Source: Infect Dis Rep. 2023 Oct 8;15(5):600–34. doi: 10.3390/idr15050058 (PMC10606787; doi:10.3390/idr15050058)
Supplement: Supplementary file 1 [file idr-15-00058-s001.zip › idr-2399682-supplementary.pdf]

Supplementary Material S1: Actual deaths versus forecast deaths in England (2001-2061)

Author RPJ has been following the gap between actuarial forecasts and actual number of deaths in the UK for over 25 years. Deaths are part of the process of estimating future population numbers, via what are called the components of change, namely births, immigration, and deaths. Figure S1 demonstrates that the forecasts for deaths are notoriously unreliable, mainly because the estimation of future mortality rates is clouded in uncertainty. The sudden change in the trajectory of total deaths after 2011 seemingly caught actuaries' completely off guard. It was only until the 2016-based forecast that they realized that the mortality rate trends had substantially changed.

Indeed, actuarial forecasts are completely unable to explain the volatility in the actual deaths, which greatly depends on infectious outbreaks each winter [40-41]. Hence official forecasts cannot be used to validate the 2019 (pre-COVID-19) base year.

It is well recognized that international estimates of COVID-19 deaths are themselves unreliable [16]. Such sources of uncertainty led to the development of several alternative methods for estimating the equivalent to a pathogen-neutral baseline for male and female single-year-of-age deaths in 2019 (the pre-COVID-19 baseline). Deaths during the COVID-19 era in 2020 and 2021 can therefore be compared against this baseline. These methods are detailed in the Supplementary files S2 to S4. While in S5 this baseline is used to determine the degree of bias in the male and female reported COVID-19 deaths in England and Wales during 2020 and 2021.

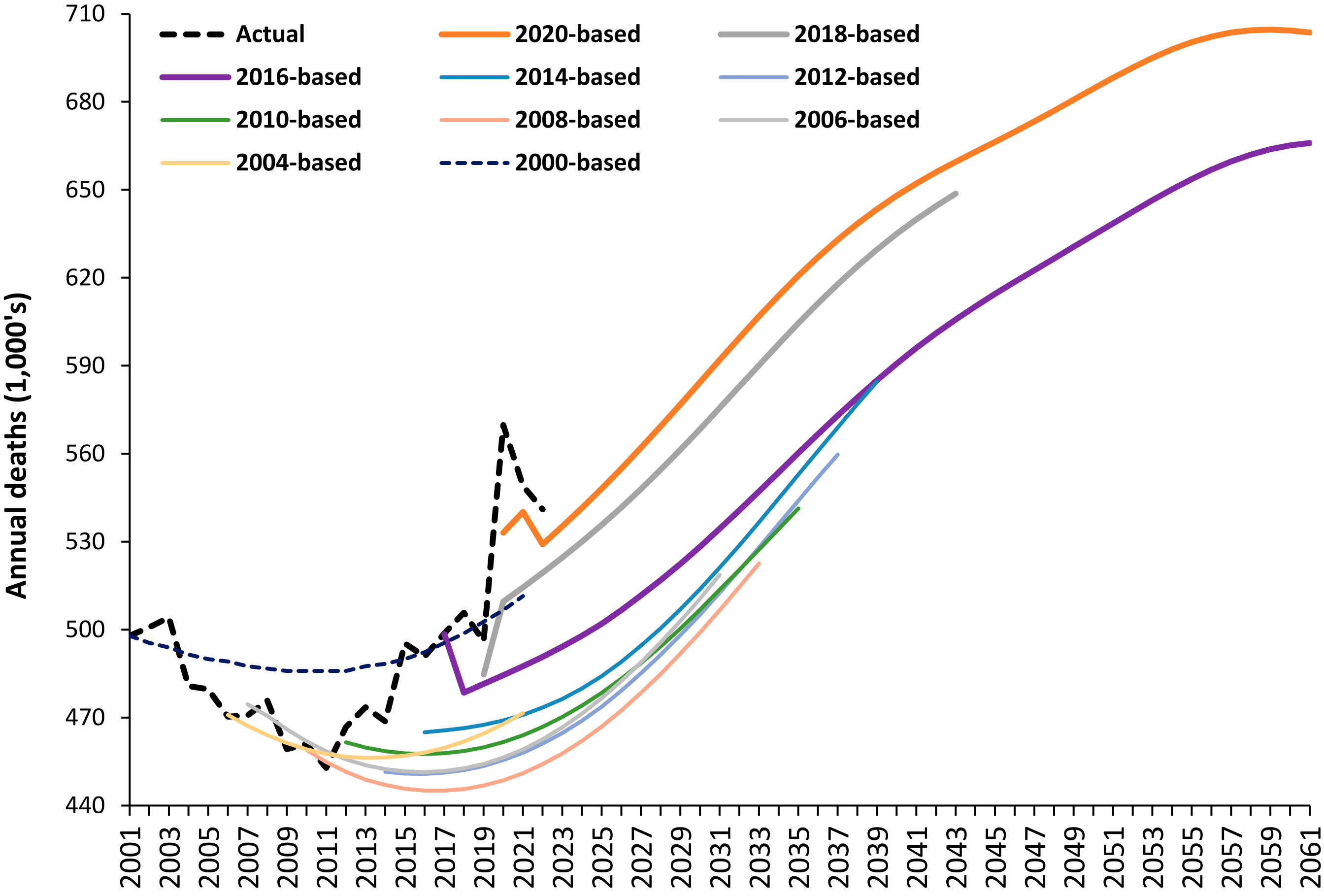

Figure S1. Actual deaths versus forecast deaths in England (2001 to 2061). Based on population forecasts prepared every two years by the Office for National Statistics (ONS) since 2000, see [38] for various past forecasts and actual deaths from [28,29].

Supplementary Material S2: Methods for adjusting deaths to the 2019 average baseline value

This is a technical Supplement relating to year of age analysis of excess deaths during the COVID-19 years from 2020 onward and how these can be adjusted to compare against a pre-COVID-19 baseline for 2019. This baseline is established as an 'average' number of deaths in 2019 based upon deaths adjusted to the 2019-equivalent in years 2011 to 2018. The resulting year of age excess deaths in 2020 and 2021 can then be compared to the measured levels of deaths 'due to' COVID-19 as assessed by the Office for National Statistics (ONS) based upon material contained in the death certificate record for each person. This assessment contains inherent diagnostic ambiguity and cannot exceed the calculated excess mortality. This comparison is made in Supplement S4.

First, we commence with an assessment of the level of year-to-year differences in deaths (or the volatility) for each year of age over the period 1963 to 2019 [from 28,29]. This is achieved by calculating the absolute value of the year-to-year difference and dividing this by the square root of the average of deaths in each pair of years. This adjusts for the effect of size and converts the absolute difference into a Poisson statistic equivalent to a standard deviation (STDEV) distance from the average. The median value for the 55 paired years for the STDEV equivalent is then calculated.

A value of the median close to 1 indicates that the volatility is driven by simple Poisson variation – as would be expected for small numbers. Values higher than 1 indicate increasing levels of special cause variation such as the impact of winter infectious outbreaks [40,41,45]. For example, an earlier study using this method established that some locations in England and Wales experience higher intrinsic volatility than others [41]. The implication being that such locations would have greater difficulty managing their budget for end-of-life and elderly care costs [41]. Note that the maximum number of male deaths of around 9500 per annum occurs at age 84, and around 11300 per annum at age 94 for females [28,29]. One STDEV of Poisson variation at the maximum deaths is therefore ± 97 (1%) for males and ± 106 (0.9%) for females.

Figure S1 shows the outcome of such analysis where the volatility is dominated by small number Poisson variation between the ages of 1 to 30 in males and 1 to 40 in females, rises above this to a maximum of around 3.1 for males aged 70 to 72 and around 3.9 for females aged 87.

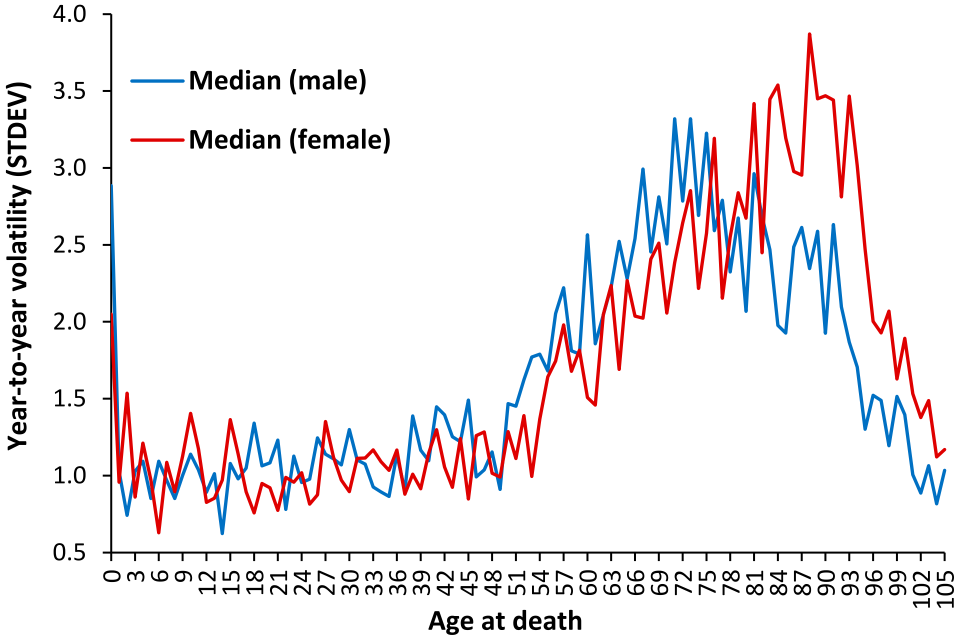

Figure S2. The median value for the year-to-year volatility in deaths (1963 to 2019).

The median volatility then declines with greater age, although above the maximum female volatility is higher. At age 0 (first year of life) volatility is higher in males. The implication of Figure S2 is that assessing the average number of deaths in 2019 (the pre-COVID-19 base year) may be more difficult at ages around those where the maximum volatility occurs – as the outworking of environmental sensitivity including infectious outbreaks.

The next steps are to calculate various adjustment factors to express all years as 2019 equivalents. Each has strengths and weaknesses.

1. Mid-year population. This method is widely used, however, both births and deaths are measured as a 12-month total and not a mid-year figure. As mentioned, the ONS does not provide mid-year population estimates above age 90 [32].
2. Based on actual births [35]. This method has the advantage that births and deaths are matched to 12-month totals. It has the disadvantage that some birth cohorts will suffer attrition in World War II and that inward/outward migration after WW II will modify the population away from the simple birth total.
3. Extrapolation based on raw single year of age deaths. This is an alternative method for age 90+ estimates. A second order polynomial is used.
4. The average of 2017, 2018 and 2019. There was a large infectious event during the winter of 2017/18 with the bulk of excess deaths occurring in 2018 [40,41,45]. This infectious event affected specific ages. Analysis shows that the excess deaths in 2018 were then counterbalanced by an almost equal number of lower deaths in both 2017 and 2019, hence, the average of the three years evens out the effect of the 2018 surge.

For population-adjusted deaths the ONS provided mid-year population estimates based on the most recent decennial census [30,31]. Since the ONS does not provide mid-year population estimates above the age of 89, the average for deaths in 2019 for ages 90+ was calculated using a second order polynomial curve fit to the raw deaths, as per #3 above.

When conducting the population adjustment, it became clear that the World War II baby boom seemed to be affecting the outcome [35]. At this point the World War I baby boom is also relevant for an alternative to the age 90+ estimated population but was also applied to ages below 90, as per #2 above.

Figure S3 therefore shows the trend in annual births over the years 1900 to 1950 [35]. Prior to World War I live births were at a maximum followed by various peaks and troughs largely emanating from World Wars I and II. The peak in deaths around 1965 arises from the WW II cohort reaching the age where births are common. The resulting adjustment factors based on live births are given in the Table in Supplement S2

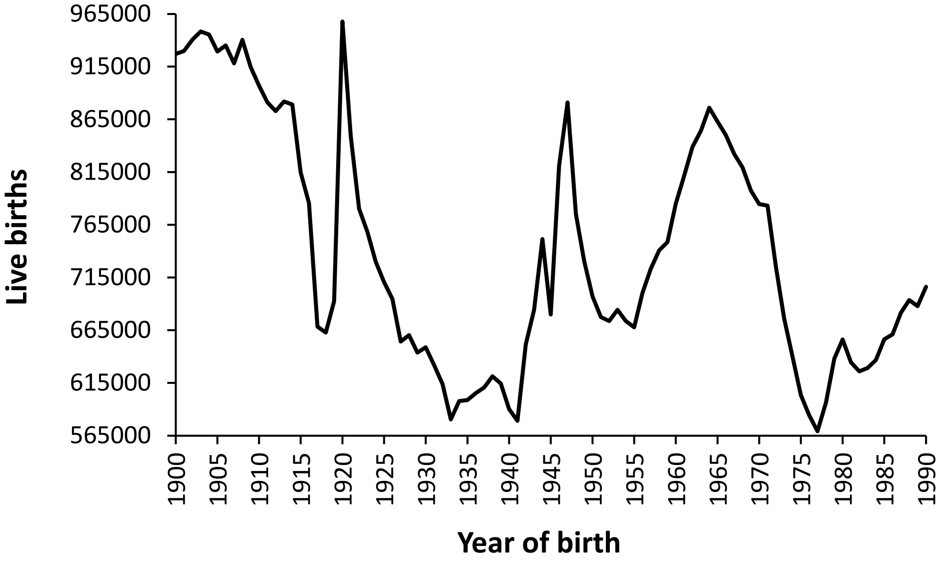

Figure S3. Trend in annual live births in England and Wales, 1900 to 1950 [35].

The major limitation of the method based on births is that those born in the WW I birth cohort suffer attrition during WW II, however, this is the only alternative to a population-based estimate.

Finally, the method based on the average of 2018 and 2019 deaths relies on the observation that the infectious event in early 2018 affected specific ages which were then approximately matched by lower deaths in these specific ages in 2019. The final chosen value for the 2019 average was therefore the average of the 3 or 4 available methods. The outcomes of the different methods are shown in Figure S4, while full details are shown in the Table given in Supplement S3.

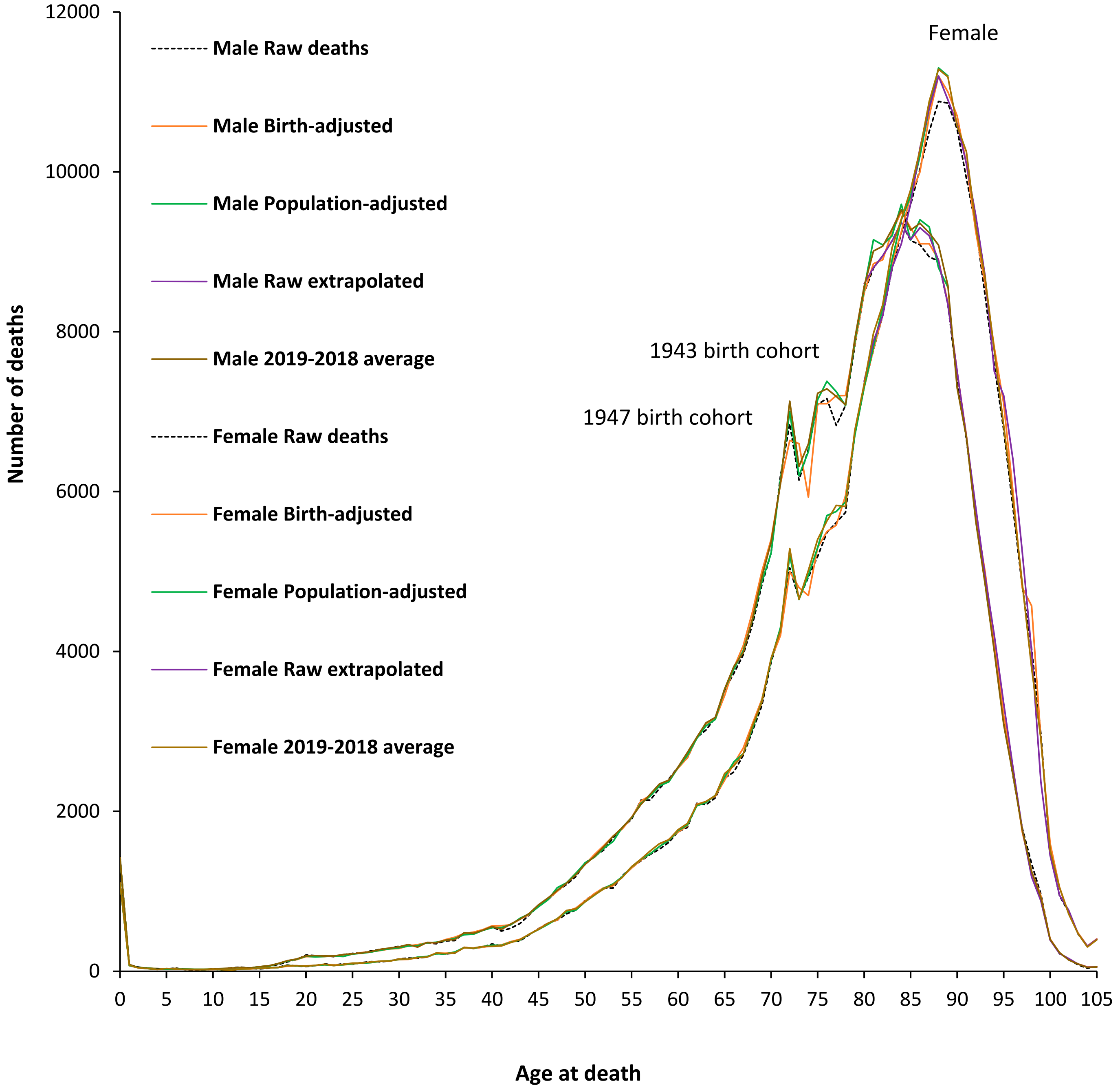

Figure S4. Results for different adjustment methods to give a 2019-average deaths baseline.

The Poisson-based variation (as a standard deviation) associated with the chosen average for 2019 was calculated as the square root of the chosen 'average' value. It should be noted that that actual calculated standard deviation between the different estimates was lower than the Poisson-based standard deviation in all years except for, Males – age 72-74, 76, 81,94-95, and Females – age 72-74, 76-77, 84,89, 94-100. This seems to be partly the outworking of issues addressed in Figure S2. Figure S5 shows the detail regarding which years have a high standard deviation between the estimates using different methods and the calculated value of a STDEV using Poisson statistics, as the square root of the estimated 'average' value.

It can be assumed that estimates for the 2019 'average' when the ratio in Figure S5 is below 100% are reasonably accurate. In the other years a degree of variation appears to be unavoidable.

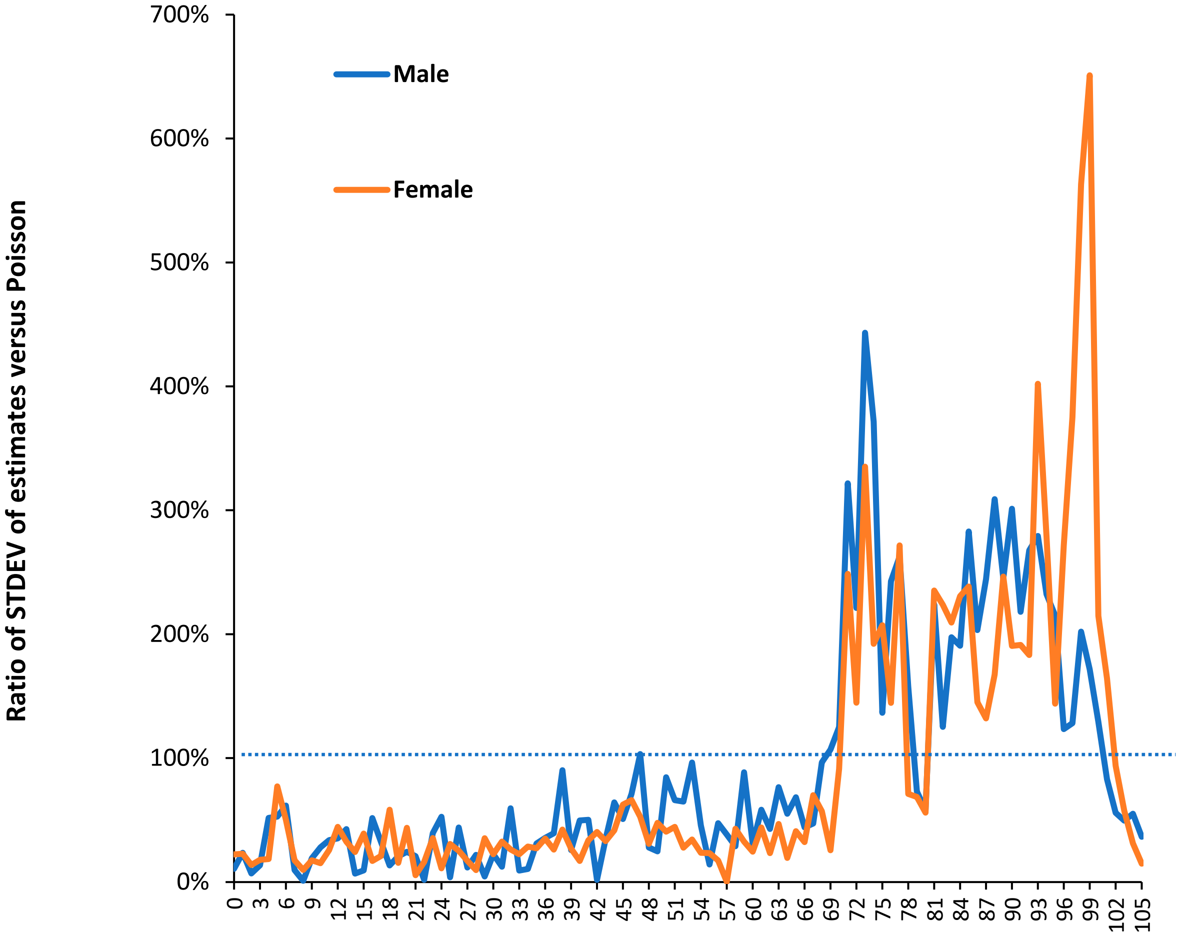

Figure S5. The ratio of the standard deviation of the estimates for 2019 versus a Poisson-based standard deviation (as the square root of the estimated 2019 'average' value.

Supplementary Material S3: Adjustment factors based on births to give 2019-equivalent estimate

Divide age data for each year by the adjustment factor to get the 2019-equivalent. Note that births reached their most recent peak in 2012. The maximum adjustment factor is 1.46 for age 92 in 2012. The minimum factor is 0.66 for age 72 in 2013. Calculation based on raw data from [35].

Age in >	2021	2020	2019	2018	2017	2016	2015	2014	2013	2012	2011
0	0.98	0.96	1.00	1.03	1.06	1.09	1.09	1.09	1.09	1.14	1.13
1	0.93	0.97	1.00	1.03	1.06	1.06	1.06	1.06	1.11	1.10	1.10
2	0.94	0.97	1.00	1.03	1.03	1.02	1.03	1.07	1.07	1.06	1.04
3	0.94	0.98	1.00	1.00	1.00	1.00	1.05	1.04	1.04	1.01	1.02
4	0.97	1.00	1.00	1.00	1.00	1.05	1.04	1.04	1.01	1.02	0.99
5	1.00	1.00	1.00	1.00	1.05	1.04	1.04	1.02	1.02	0.99	0.96
6	1.00	1.00	1.00	1.04	1.04	1.04	1.01	1.01	0.99	0.96	0.92
7	0.95	0.96	1.00	0.99	0.99	0.97	0.97	0.95	0.92	0.89	0.88
8	0.96	1.01	1.00	1.00	0.98	0.98	0.95	0.92	0.89	0.88	0.86
9	1.01	1.00	1.00	0.98	0.98	0.95	0.93	0.89	0.88	0.86	0.82
10	1.03	1.02	1.00	1.00	0.98	0.95	0.91	0.91	0.88	0.84	0.84
11	1.02	1.00	1.00	0.97	0.94	0.91	0.90	0.88	0.84	0.84	0.85
12	1.02	1.03	1.00	0.97	0.94	0.93	0.90	0.86	0.86	0.88	0.90
13	1.06	1.03	1.00	0.96	0.96	0.93	0.89	0.89	0.90	0.93	0.95
14	1.07	1.04	1.00	0.99	0.96	0.92	0.92	0.94	0.96	0.98	1.00
15	1.05	1.01	1.00	0.97	0.93	0.93	0.94	0.97	0.99	1.01	1.02
16	1.04	1.03	1.00	0.96	0.96	0.97	1.00	1.02	1.03	1.05	1.04
17	1.07	1.04	1.00	1.00	1.01	1.04	1.07	1.08	1.09	1.09	1.12
18	1.05	1.00	1.00	1.02	1.05	1.07	1.08	1.09	1.09	1.12	1.13
19	0.99	0.98	1.00	1.03	1.05	1.06	1.07	1.07	1.10	1.11	1.14
20	0.96	0.97	1.00	1.02	1.03	1.04	1.04	1.07	1.08	1.11	1.12
21	0.95	0.98	1.00	1.01	1.02	1.02	1.05	1.06	1.08	1.10	1.11
22	0.97	0.99	1.00	1.01	1.01	1.03	1.05	1.07	1.09	1.10	1.07
23	0.98	0.99	1.00	1.00	1.02	1.04	1.06	1.08	1.09	1.06	1.07
24	0.99	1.00	1.00	1.03	1.04	1.06	1.08	1.09	1.06	1.07	1.05
25	0.98	0.98	1.00	1.01	1.04	1.05	1.06	1.03	1.04	1.03	0.99
26	0.96	0.99	1.00	1.02	1.04	1.05	1.02	1.03	1.01	0.98	0.97
27	0.96	0.98	1.00	1.01	1.02	1.00	1.01	0.99	0.96	0.95	0.92
28	0.96	0.99	1.00	1.01	0.98	0.99	0.97	0.95	0.94	0.91	0.90
29	0.98	0.99	1.00	0.97	0.98	0.97	0.94	0.93	0.90	0.89	0.89
30	1.02	1.03	1.00	1.01	0.99	0.96	0.95	0.93	0.91	0.91	0.92
31	1.02	0.99	1.00	0.98	0.95	0.95	0.92	0.91	0.90	0.91	0.95
32	1.01	1.02	1.00	0.97	0.96	0.93	0.92	0.92	0.93	0.96	0.94
33	1.05	1.03	1.00	0.99	0.96	0.95	0.95	0.96	0.99	0.97	0.90
34	1.04	1.01	1.00	0.97	0.96	0.95	0.97	1.00	0.97	0.91	0.87
35	1.04	1.03	1.00	0.99	0.98	1.00	1.03	1.00	0.94	0.89	0.92
36	1.04	1.01	1.00	0.99	1.01	1.04	1.01	0.95	0.90	0.93	0.96
37	1.02	1.01	1.00	1.01	1.05	1.02	0.95	0.91	0.93	0.96	1.02
38	0.99	0.99	1.00	1.03	1.01	0.94	0.90	0.92	0.95	1.01	1.07
39	0.95	0.97	1.00	0.97	0.91	0.87	0.89	0.92	0.98	1.03	1.11
40	0.99	1.03	1.00	0.93	0.89	0.92	0.95	1.00	1.06	1.14	1.23
41	1.10	1.07	1.00	0.95	0.98	1.01	1.07	1.13	1.22	1.31	1.32
42	1.12	1.05	1.00	1.03	1.06	1.12	1.19	1.27	1.38	1.38	1.40

Age in >	2021	2020	2019	2018	2017	2016	2015	2014	2013	2012	2011
43	1.02	0.97	1.00	1.03	1.10	1.16	1.24	1.34	1.34	1.37	1.40
44	0.94	0.97	1.00	1.06	1.12	1.20	1.30	1.30	1.32	1.36	1.38
45	0.91	0.94	1.00	1.06	1.13	1.22	1.23	1.25	1.28	1.30	1.33
46	0.89	0.95	1.00	1.07	1.16	1.16	1.18	1.21	1.23	1.26	1.28
47	0.88	0.93	1.00	1.08	1.08	1.10	1.13	1.15	1.17	1.19	1.21
48	0.86	0.93	1.00	1.00	1.02	1.05	1.06	1.09	1.10	1.12	1.09
49	0.92	1.00	1.00	1.02	1.04	1.06	1.08	1.10	1.12	1.09	1.07
50	0.98	0.98	1.00	1.03	1.04	1.07	1.08	1.10	1.07	1.05	1.02
51	0.96	0.97	1.00	1.02	1.04	1.05	1.07	1.04	1.02	0.99	0.96
52	0.96	0.98	1.00	1.02	1.04	1.05	1.03	1.01	0.97	0.94	0.90
53	0.96	0.98	1.00	1.02	1.03	1.00	0.99	0.95	0.92	0.88	0.87
54	0.96	0.99	1.00	1.02	0.99	0.97	0.94	0.91	0.87	0.86	0.84
55	0.97	0.98	1.00	0.97	0.96	0.93	0.90	0.85	0.85	0.83	0.80
56	1.01	1.03	1.00	0.98	0.95	0.92	0.88	0.87	0.85	0.82	0.78
57	1.04	1.02	1.00	0.97	0.94	0.89	0.88	0.86	0.83	0.80	0.80
58	1.05	1.03	1.00	0.97	0.92	0.91	0.89	0.86	0.82	0.83	0.84
59	1.07	1.03	1.00	0.95	0.94	0.92	0.89	0.85	0.86	0.87	0.86
60	1.08	1.05	1.00	0.99	0.97	0.94	0.89	0.90	0.91	0.90	0.91
61	1.06	1.01	1.00	0.98	0.95	0.90	0.91	0.92	0.91	0.91	0.94
62	1.03	1.02	1.00	0.97	0.92	0.93	0.95	0.93	0.94	0.96	1.01
63	1.06	1.03	1.00	0.95	0.96	0.98	0.96	0.97	1.00	1.04	1.11
64	1.08	1.05	1.00	1.01	1.02	1.01	1.01	1.04	1.09	1.16	1.32
65	1.04	0.99	1.00	1.02	1.00	1.01	1.03	1.08	1.15	1.31	1.22
66	0.98	0.98	1.00	0.98	0.99	1.02	1.07	1.13	1.29	1.20	0.99
67	1.00	1.02	1.00	1.01	1.03	1.08	1.15	1.31	1.22	1.01	1.12
68	1.01	0.99	1.00	1.03	1.08	1.14	1.30	1.21	1.00	1.11	1.01
69	0.97	0.97	1.00	1.05	1.11	1.26	1.18	0.98	1.08	0.98	0.93
70	0.93	0.95	1.00	1.06	1.21	1.12	0.93	1.03	0.94	0.89	0.79
71	0.90	0.94	1.00	1.14	1.06	0.88	0.97	0.88	0.84	0.75	0.76
72	0.83	0.88	1.00	0.93	0.77	0.85	0.78	0.74	0.66	0.67	0.70
73	0.94	1.07	1.00	0.83	0.92	0.83	0.79	0.71	0.72	0.75	0.76
74	1.30	1.21	1.00	1.11	1.01	0.96	0.85	0.87	0.90	0.91	0.90
75	1.09	0.90	1.00	0.91	0.87	0.77	0.79	0.82	0.83	0.81	0.81
76	0.99	1.10	1.00	0.95	0.85	0.86	0.90	0.91	0.89	0.88	0.87
77	1.15	1.05	1.00	0.89	0.91	0.94	0.95	0.94	0.93	0.92	0.92
78	1.18	1.13	1.00	1.02	1.06	1.07	1.05	1.05	1.03	1.03	1.00
79	1.10	0.98	1.00	1.04	1.05	1.03	1.03	1.01	1.01	0.98	1.04
80	0.94	0.96	1.00	1.01	0.99	0.99	0.97	0.97	0.94	1.00	1.03
81	0.95	0.99	1.00	0.98	0.97	0.96	0.96	0.93	0.99	1.02	1.04
82	1.01	1.02	1.00	0.99	0.98	0.98	0.95	1.01	1.04	1.06	1.05
83	1.03	1.01	1.00	0.99	0.99	0.96	1.01	1.04	1.07	1.06	1.09
84	1.02	1.01	1.00	1.00	0.97	1.03	1.06	1.08	1.08	1.10	1.09
85	1.01	1.00	1.00	0.97	1.03	1.06	1.09	1.08	1.10	1.09	1.16
86	1.03	1.03	1.00	1.06	1.09	1.12	1.11	1.14	1.13	1.20	1.22
87	0.97	0.95	1.00	1.03	1.06	1.05	1.08	1.07	1.13	1.16	1.19
88	0.92	0.97	1.00	1.03	1.02	1.04	1.03	1.10	1.12	1.15	1.20
89	0.95	0.97	1.00	0.99	1.02	1.01	1.07	1.10	1.13	1.17	1.20
90	0.98	1.01	1.00	1.03	1.02	1.08	1.10	1.13	1.18	1.21	1.32
91	0.98	0.97	1.00	0.99	1.05	1.08	1.11	1.15	1.18	1.29	1.45

Age in >	2021	2020	2019	2018	2017	2016	2015	2014	2013	2012	2011
92	0.98	1.01	1.00	1.06	1.09	1.12	1.16	1.19	1.30	1.46	1.06
93	0.95	0.94	1.00	1.02	1.05	1.09	1.12	1.22	1.38	1.00	0.95
94	0.92	0.98	1.00	1.03	1.07	1.10	1.19	1.35	0.97	0.93	0.94
95	0.95	0.97	1.00	1.04	1.07	1.16	1.31	0.95	0.91	0.92	1.08
96	0.94	0.96	1.00	1.03	1.12	1.26	0.91	0.87	0.88	1.04	1.07
97	0.94	0.97	1.00	1.09	1.23	0.89	0.85	0.86	1.01	1.04	1.13
98	0.89	0.92	1.00	1.13	0.82	0.78	0.79	0.93	0.96	1.04	1.04
99	0.81	0.89	1.00	0.72	0.69	0.70	0.82	0.85	0.92	0.92	0.91
100	1.23	1.38	1.00	0.96	0.97	1.13	1.18	1.27	1.27	1.26	1.27
101	1.45	1.04	1.00	1.01	1.19	1.23	1.33	1.33	1.32	1.33	1.35
102	1.04	0.99	1.00	1.18	1.22	1.32	1.32	1.31	1.32	1.34	1.37
103	0.84	0.85	1.00	1.04	1.12	1.12	1.11	1.12	1.14	1.16	1.20
104	0.82	0.96	1.00	1.08	1.08	1.07	1.08	1.10	1.12	1.15	1.13
105	0.89	0.93	1.00	1.00	0.99	1.00	1.02	1.04	1.07	1.04	1.06

Supplementary Material S4: Results for the alternative methods to calculate the 2019 'baseline' number of deaths. The raw deaths in 2019 are provided for comparison. A Poisson-based standard deviation (STDEV) based on the chosen 'average' value for 2019 is also given as is the STDEV of the estimates. Population-adjusted deaths end at age 89. Raw extrapolated deaths commence at age 80. Hence ages 80-89 are the average of 5 methods while all other ages are an average of 3 methods. Method Birth Adjusted (B) assumes that above the age of 80 the impact of COVID-19 on genuine COVID-19 deaths is minimal and so all years 2011 to 2021 are included in the estimate for the 2019 baseline.

Age	Male	Male	Male	Male	Male	Male	Male	Male	Male	Female	Female	Female	Female	Female	Female	Female	Female	Female
	Raw deaths	Birth-adjusted (A)	Birth-adjusted (B)	Population-adjusted	Raw extrapolated	2019-2017 average	Chosen value	STDEV Poisson	STDEV raw	Raw deaths	Birth-adjusted (A)	Birth-adjusted (B)	Population-adjusted	Raw extrapolated	2019-2017 average	Chosen value	STDEV Poisson	STDEV raw
0	1435	1420		1415		1423	1419	38	4	1119	1100		1104		1114	1106	33	7
1	79	80		80		84	81	9	2	78	68		72		71	70	8	2
2	49	48		48		47	48	7	0	51	42		44		43	43	7	1
3	33	40		39		38	39	6	1	34	32		33		34	33	6	1
4	27	30		30		35	32	6	3	27	23		23		25	24	5	1
5	30	27		28		32	29	5	3	31	23		29		31	27	5	4
6	40	27		33		33	31	6	3	33	23		27		28	26	5	3
7	21	28		27		28	28	5	1	21	23		25		23	24	5	1
8	19	25		25		25	25	5	0	19	22		22		23	22	5	0
9	19	25		25		23	24	5	1	18	20		21		20	20	5	1
10	27	25		27		28	27	5	1	26	22		23		22	23	5	1
11	30	28		28		31	29	5	2	17	23		21		22	22	5	1
12	43	35		38		39	37	6	2	13	20		17		20	19	4	2
13	51	39		41		44	41	6	3	32	30		29		27	29	5	2
14	36	40		40		39	40	6	0	34	30		33		30	31	6	1
15	45	58		57		58	58	8	1	30	38		36		33	36	6	2
16	66	66		65		73	68	8	4	40	44		46		45	45	7	1
17	83	95		94		100	96	10	3	50	45		46		48	46	7	1
18	117	129		127		130	129	11	2	76	63		73		68	68	8	5
19	147	145		150		147	147	12	3	66	65		67		66	66	8	1

20	202	182	187	181	183	14	3	59	68	63	61	64	8	3
21	196	184	180	185	183	14	3	70	72	71	72	72	8	0
22	192	184	184	184	184	14	0	91	80	82	80	81	9	1
23	182	193	190	182	188	14	5	76	78	72	73	74	9	3
24	207	197	185	198	193	14	7	93	80	82	80	81	9	1
25	223	216	215	215	215	15	1	86	90	94	96	93	10	3
26	229	233	225	220	226	15	7	108	105	102	100	102	10	3
27	255	243	240	240	241	16	2	121	110	107	111	109	10	2
28	274	260	265	267	264	16	4	118	125	125	123	124	11	1
29	288	279	280	280	280	17	1	129	130	128	122	127	11	4
30	313	286	293	288	289	17	4	154	145	149	144	146	12	3
31	316	317	317	321	318	18	2	165	148	152	156	152	12	4
32	319	333	320	312	322	18	11	162	170	175	176	174	13	3
33	354	355	355	352	354	19	2	179	180	184	186	183	14	3
34	344	358	362	359	360	19	2	220	225	218	217	220	15	4
35	380	397	385	389	390	20	6	217	225	217	221	221	15	4
36	383	425	412	413	417	20	7	227	230	241	234	235	15	5
37	478	472	456	470	466	22	8	298	300	295	291	296	17	4
38	482	490	463	452	468	22	20	285	285	287	274	282	17	7
39	516	520	510	509	513	23	6	304	300	309	307	305	17	5
40	556	568	545	554	556	24	12	341	320	326	324	323	18	3
41	503	570	547	555	557	24	12	316	330	323	335	329	18	6
42	538	580	580	581	580	24	0	367	370	362	355	362	19	8
43	597	650	662	644	652	26	9	380	400	390	388	393	20	7
44	705	710	717	685	704	27	17	457	460	464	447	457	21	9
45	819	830	807	803	813	29	14	534	530	522	502	518	23	14
46	894	900	891	860	884	30	21	600	600	586	568	585	24	16
47	1005	1000	1045	982	1009	32	33	640	640	657	631	643	25	13
48	1084	1100	1105	1087	1097	33	9	717	750	743	733	742	27	8
49	1184	1226	1224	1210	1220	35	9	765	790	764	773	776	28	13
50	1354	1335	1357	1296	1329	36	31	887	880	869	856	868	29	12
51	1440	1460	1425	1412	1432	38	25	967	970	950	944	955	31	14
52	1515	1570	1536	1520	1542	39	25	1042	1045	1036	1027	1036	32	9
53	1668	1690	1622	1623	1645	41	39	1041	1070	1092	1080	1081	33	11
54	1797	1780	1800	1761	1780	42	19	1195	1180	1182	1167	1176	34	8
55	1899	1920	1918	1908	1915	44	6	1296	1290	1305	1304	1300	36	8

56	2141	2130		2100		2088	2106	46	22	1382	1390		1400		1388	1393	37	6
57	2141	2200		2190		2225	2205	47	18	1461	1470		1470		1470	1470	38	0
58	2288	2330		2318		2346	2331	48	14	1527	1560		1560		1589	1570	40	17
59	2405	2390		2370		2453	2404	49	43	1613	1650		1635		1661	1649	41	13
60	2553	2540		2550		2570	2553	51	15	1744	1740		1760		1747	1749	42	10
61	2667	2670		2700		2731	2700	52	30	1799	1820		1830		1856	1835	43	19
62	2923	2920		2910		2954	2928	54	23	2100	2090		2070		2074	2078	46	11
63	3019	3100		3070		3154	3108	56	43	2082	2120		2110		2151	2127	46	22
64	3171	3150		3150		3204	3168	56	31	2168	2190		2200		2208	2199	47	9
65	3517	3450		3530		3480	3487	59	40	2415	2390		2430		2406	2409	49	20
66	3728	3800		3780		3834	3805	62	27	2493	2600		2620		2587	2602	51	16
67	3957	4070		4010		4041	4040	64	30	2701	2790		2720		2775	2762	53	37
68	4344	4500		4400		4380	4427	67	64	3007	3100		3040		3053	3064	55	32
69	4832	5000		4870		4871	4914	70	75	3321	3400		3377		3372	3383	58	15
70	5232	5400		5230		5259	5296	73	91	3865	3900		3890		3797	3862	62	57
71	6195	6100		6150		5696	5982	77	249	4256	4200		4300		3986	4162	65	160
72	6847	6640		7000		6876	6839	83	183	5045	5000		5200		5056	5085	71	103
73	6144	6600		6200		6917	6572	81	359	4662	4800		4650		5108	4853	70	233
74	6529	5930		6500		6339	6256	79	294	4929	4700		4950		4746	4799	69	133
75	7092	7100		7150		7321	7190	85	116	5188	5300		5300		5563	5388	73	152
76	7163	7100	7600	7380		7286	7342	86	208	5489	5500		5700		5672	5624	75	108
77	6828	7200	7700	7250		7425	7394	86	226	5613	5580	6000	5751		6008	5835	76	207
78	7078	7200	7400	7075		7216	7223	85	134	5740	5950	6000	5870		5963	5946	77	55
79	7822	7840	7900	7875		7752	7842	89	65	6726	6720	6700	6700		6596	6679	82	56
80	8489	8490	8600	8540	8600	8496	8545	92	54	7312	7300	7400	7307	7380	7304	7338	86	48
81	8795	8850	9300	9150	8800	8922	9004	95	213	7771	7780	8300	7810	7880	7931	7940	89	210
82	8951	8900	9200	9085	8950	8992	9025	95	119	8204	8210	8700	8300	8200	8343	8351	91	204
83	9137	9250	9600	9200	9135	9160	9269	96	190	8787	8850	9300	8900	8800	8949	8960	95	198
84	9364	9500	9800	9595	9370	9343	9522	98	186	9237	9250	9700	9400	9100	9421	9374	97	223
85	9141	9300	9800	9145	9150	9229	9325	97	273	9588	9700	10200	9700	9600	9862	9812	99	236
86	9087	9100	9300	9400	9300	8913	9203	96	195	10037	10000	10300	10200	10300	10021	10164	101	146
87	8935	9100	9500	9310	9200	8872	9196	96	234	10505	10700	11000	10800	10800	10639	10788	104	137
88	8883	8900	9400	8800	8890	8613	8921	94	292	10882	11200	11500	11300	11200	11013	11243	106	177
89	8340	8360	8700	8555	8340	8111	8413	92	225	10860	11000	11500	11200	10900	10877	11095	105	260
90	7405	7500	7900		7500	7271	7543	87	262	10530	10700	11000		10600	10566	10717	104	197
91	6655	6700	7000		6685	6585	6743	82	179	9917	10100	10500		10100	10148	10212	101	193

92	5725	5700	6000	5798	5513	5753	76	203	9318	9250	9600	9450	9224	9381	97	177
93	4950	5000	5200	4950	4721	4968	70	197	8453	8600	9400	8700	8646	8836	94	378
94	4081	4110	4110	4200	3856	4069	64	148	7574	7800	8100	7500	7754	7788	88	246
95	3159	3200	3300	3350	3073	3231	57	122	6769	7100	7042	7200	6910	7063	84	121
96	2449	2540	2530	2550	2417	2509	50	62	5799	6000	6000	6400	5939	6085	78	212
97	1792	1745	1800	1780	1678	1751	42	54	4814	4800	4900	5250	4627	4894	70	263
98	1346	1250	1330	1180	1182	1235	35	71	4032	4570	4122	4000	3702	4098	64	360
99	964	943	1000	880	904	932	31	52	2971	2900	3200	2370	2936	2851	53	348
100	398	405	450	390	403	412	20	26	1475	1600	1650	1450	1574	1568	40	85
101	221	230	250	222	225	232	15	13	944	1050	1070	950	1029	1025	32	53
102	151	145	145	158	143	148	12	7	740	720	705	760	709	723	27	25
103	80	92	92	89	82	89	9	5	466	480	500	470	487	484	22	13
104	37	54	54	51	46	51	7	4	309	315	320	316	307	314	18	6
105	61	57	58	53	53	55	7	3	399	405	405	399	401	403	20	3

Supplementary Material S5: Deaths assessed as 'due to' COVID-19 in 2020 and 2021 compared to excess deaths relative to 2019

Deaths assessed as 'due to' COVID-19 in 2020 and 2021 cannot be higher than the excess deaths occurring in these years relative to the 2019 baseline. Some have claimed that lockdowns led to excess deaths [46]. Based on this claim excess deaths should logically be higher than deaths assessed as 'due to' COVID-19 [47].

Table S1 shows the total 'excess' or 'extra' deaths in England and Wales in both 2022 (without vaccination) and 2021 (with vaccination) compared to the deaths which were assessed as 'due to' COVID-19. The 'excess' deaths are given for both:

1. 2020 or 2021 actual minus 2019 'baseline'
2. 2020 or 2021 actual minus 2019 actual

In this comparison recall that for the trend between 2011 and 2019, 2019 was assessed as a low year against the trend due to a large excess of winter deaths at the start of 2018 which led to a compensating drop in deaths during 2019. Hence, method #1 will give a lower estimate for the 'excess' deaths.

In 2020 (without vaccination) assessed 'due to' COVID-19 deaths are very close to the calculated 'excess', however, in 2021 (with vaccination) there is a very large discrepancy where so-called 'due to' COVID-19 deaths are far higher than is mathematically possible. This discrepancy is far greater for female deaths. Indeed, regarding the issue of over counting deaths in England and Wales assessed as 'with' COVID-19 are another 15% higher than the assessed 'due to' deaths.

Table S1. Evaluation of potential over counting of 'due to' COVID-19 deaths in 2020 and 2021.

Method	Sex/Year	Calculated excess	ONS 'due to'	Over estimate (n)	Over estimate (%)
Actual minus 2019 'average'	Male 2021	30 002	36 794	6 792	23%
	Male 2020	40 082	40 995	913	2%
	Female 2021	18 816	30 556	11 740	62%
	Female 2020	30 324	32 771	2447	8%
Actual minus 2019 actual	Male 2021	32 689	36 794	4 105	13%
	Male 2020	42 769	40 995	-1 774	-4%
	Female 2021	22 804	30 556	7 752	34%
	Female 2020	34 312	32 771	-1 541	-4%

The issue as to which age bands were more prone to over counting is addressed in Table S2. As can be seen during 2020 (without vaccination) the highest over counting is limited to ages 65-69 and 80-89 (with yellow highlight), with highest over counting at age 65-69 at +19% males and + 40% females.

In contrast, the degree of over estimation in 2021 (with vaccination) is greatest at ages 65-74 and for all ages above 80 years. Female over counting is greatest in the 85-89 age band at +353%. Such over estimation during the with vaccination period may reflect the moderate ability of COVID-19 vaccines to prevent infection per se [48,49], although viral load is reduced [50].

Table S2. Evaluation of potential over counting of 'due to' COVID-19 deaths in 2020 and 2021 for males and females by age band.

Year	Age group	Males 'due to'	Females 'due to'	Male excess	Female excess	Male overestimate (n)	Female overestimate (n)	Male overestimate (%)	Female overestimate (%)
2021	*All ages*	*36794*	*30556*	*30002*	*18816*	*6792*	*11740*	*23%*	*62%*
2021	Under 1	2	0	-3	-20	5	20		
2021	1 to 4	2	3	-24	-28	26	31		
2021	5 to 9	1	6	-18	-16	19	22		
2021	10 to 14	3	9	-34	7	37	2		
2021	15 to 19	18	11	81	-14	-63	25		
2021	20 to 24	32	15	-2	35	34	-20		
2021	25 to 29	55	40	1	28	54	12		
2021	30 to 34	133	85	129	95	4	-10		
2021	35 to 39	216	147	353	207	-137	-60		
2021	40 to 44	366	208	649	348	-283	-140		
2021	45 to 49	622	369	618	244	4	125	1%	51%
2021	50 to 54	1038	652	1342	614	-304	38	-23%	6%
2021	55 to 59	1678	982	2105	1167	-427	-185	-20%	-16%
2021	60 to 64	2428	1354	3260	1588	-832	-234	-26%	-15%
2021	65 to 69	2965	1847	2170	1303	795	544	37%	42%
2021	70 to 74	4195	2642	3484	2260	711	382	20%	17%
2021	75 to 79	5168	3417	5616	3828	-448	-411	-8%	-11%
2021	80 to 84	6177	4775	3218	2434	2959	2341	92%	96%
2021	85 to 89	6177	5815	2846	1283	3331	4532	117%	353%
2021	90+	5518	8179	4213	3454	1305	4725	31%	137%
2020	*All ages*	*40995*	*32771*	*40082*	*30324*	*913*	*2447*	*2%*	*8%*
2020	Under 1	0	0	-84	-56	84	56		
2020	1 to 4	0	0	-13	-31	13	31		
2020	5 to 9	0	1	8	-45	-8	46		
2020	10 to 14	3	1	-21	4	24	-3		
2020	15 to 19	5	5	-60	-28	65	33		
2020	20 to 24	16	12	-96	-42	112	54		
2020	25 to 29	44	25	-71	-28	115	53		
2020	30 to 34	67	46	-7	43	74	3		
2020	35 to 39	98	76	168	143	-70	-67		
2020	40 to 44	197	132	326	227	-129	-95		
2020	45 to 49	386	233	465	250	-79	-17	-17%	-7%
2020	50 to 54	724	423	1015	484	-291	-61	-29%	-13%
2020	55 to 59	1277	666	1817	726	-540	-60	-30%	-8%
2020	60 to 64	1966	990	2456	1206	-490	-216	-20%	-18%
2020	65 to 69	2730	1412	2289	1008	441	404	19%	40%
2020	70 to 74	4462	2426	4475	2409	-13	17	0%	1%
2020	75 to 79	6229	3823	6385	4086	-156	-263	-2%	-6%
2020	80 to 84	8158	5891	7192	5258	966	633	13%	12%
2020	85 to 89	7980	7159	6989	5420	991	1739	14%	32%
2020	90+	6653	9450	6851	9291	-198	159	-3%	2%

There is a simple explanation for the increased over counting in 2021. Toward the end of 2020 COVID-19 testing capacity was greatly expanded [14] resulting in many more persons testing positive. This led to an expansion in the number of persons who died

'with' COVID-19. Ambiguity regarding the true cause of death then led to many of these 'with' COVID-19 deaths being incorrectly coded as 'due to' COVID-19. See below for a more comprehensive view using data from Scotland.

Figure A5 which is a 'with' COVID-19 view can therefore be correctly interpreted as being a large overestimate of the real situation from late 2020 onward.

However, limitations aside, available evidence must be used within a knowledge of unavoidable ambiguity.

Regarding the issue of unavoidable ambiguity Figure S6 shows the assessed 'due to' ('underlying cause' in Scotland) and 'with' ('mentioned' in Scotland) COVID-19 deaths in Scotland. Note that Scotland has a different death certification process to England and Wales and may be more realistic than the former.

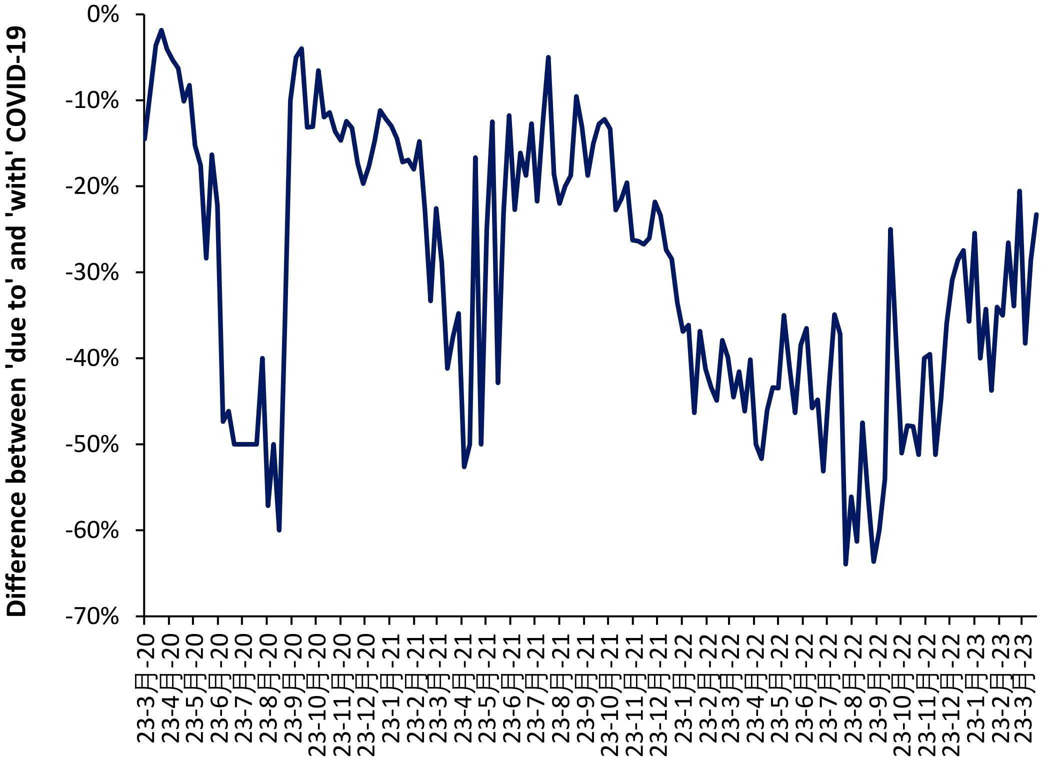

Figure S6. Difference between the number of assessed 'with or mentioned' COVID-19 and 'due to or underlying cause' COVID-19 deaths in Scotland from March 2020 to March 2023. Data is from Table 9 in [51].

From the Scottish data it is evident that the gap between the two measures of COVID-19 death is dependent on the variant and the active phase of any outbreak. Hence in July and August 2020 when the original Wuhan variant wave had fallen to a minimum there is a large gap between the two. The gap then narrows as the second wave rises and is replaced by the Alpha variant. Another large gap ensues as the Alpha wave falls to a minimum. The same applies to the Delta wave. Omicron is far more nuanced since it behaves as an endemic disease – see Figure A5 in the Appendix of the main document. However, after the initial Omicron outbreak, the gap then appears to diminish with time. Assessing the number of deaths where COVID-19 is the underlying cause is less than simple, and even 'with' COVID-19 deaths can depend on the level of available testing capacity.

Hence, the primary reason for our emphasis on excess mortality in this study – which is independent of the vagaries of assigning cause of death.
